# FBXO31 is upregulated by METTL3 to promote pancreatic cancer progression via regulating SIRT2 ubiquitination and degradation

**DOI:** 10.1038/s41419-024-06425-y

**Published:** 2024-01-12

**Authors:** Kai Chen, Yue Wang, Xingna Dai, Jingjing Luo, Shangshang Hu, Zhihui Zhou, Jinglong Shi, Xueshan Pan, Tong Cao, Jun Xia, Yuyun Li, Zhiwei Wang, Jia Ma

**Affiliations:** 1 Bengbu Medical University Key Laboratory of Cancer Research and Clinical Laboratory Diagnosis, Bengbu Medical University, Anhui Bengbu, 233030 China; 2 Department of Laboratory Medicine, School of Laboratory Medicine, Bengbu Medical University, Anhui Bengbu, 233030 China; 3 Department of Biochemistry and Molecular Biology, School of Laboratory Medicine, Bengbu Medical University, Anhui Bengbu, 233030 China; 4 Department of Clinical Laboratory, the First Affiliated Hospital of Bengbu Medical University, Anhui Bengbu, 233004 China; 5 Department of Clinical Laboratory Diagnostics, School of Laboratory Medicine, Bengbu Medical University, Anhui Bengbu, 233030 China

**Keywords:** Pancreatic cancer, Ubiquitylation

## Abstract

FBXO31, a member of F-box family to comprise of SCF complex, contributes to a pivotal role in cancer progression. However, the possible involvements of FBXO31 in PC are unelucidated. Here, we reported that FBXO31 was overexpressed in PC patients, which was negatively associated with survival in PC patients. Furthermore, FBXO31 significantly enhanced growth, migration and invasion of PC cells in vitro. Consistently, FBXO31 overexpression promoted tumor growth in nude mice. Mechanistically, SIRT2 was a target of FBXO31 and interacted with FBXO31. Protein half-life and ubiquitination analysis demonstrated that FBXO31 promoted proteasome-dependent degradation of SIRT2. In addition, FBXO31 binds to sirtuin-type domain of SIRT2. Moreover, SIRT2 is required for the oncogenic role of FBXO31 in PC progression. Impressively, METTL3 induced m6A modification of FBXO31 and up-regulated FBXO31 expression, subsequently leading to SIRT2 down-regulation in PC cells. The results showed that METTL3 enhanced FBXO31 mRNA translation in YTHDF1-dependent manner. Taken together, we suggest that METTL3–FBXO31–SIRT2 axis was involved in PC tumorigenesis, which could identify new targets for PC treatment.

## Introduction

Pancreatic cancer (PC) is one of most lethal human carcinomas worldwide, which accounts for its lowest 5-year survival rate about 8% [1, 2]. Furthermore, PC incidence continuously increases in both men and women in the United States, which is estimated approximately 64,050 new cases in 2023 [3]. As possession of high aggressiveness and hidden symptoms, only 10–20% of PC patients are localized disease at diagnosis. PC patients are often treated with surgical resection and subsequently adjuvant systemic chemotherapy [4]. And 5-year survival rate of surgical resectable PC patients only reaches to 30% after surgery and adjuvant chemotherapy treatments [4]. Thus, it is urgent to comprehensively understand the molecular mechanisms of PC and develop novel strategies for treatment of PC.

F-box protein FBXO31, functioning as the substrate recognition protein of SCF (SKP/Cullin/F-box protein) class E3 ubiquitin ligase, is responsible for mediating substrate ubiquitination and degradation [5, 6]. FBXO31 was identified as a tumor suppressor in breast cancer and prostate cancers and it located at chromosome 16q24.3 loss of heterozygosity region [7, 8]. It has been suggested that FBXO31 plays a key role in biological process of DNA repair, cell cycle, cell growth and metastasis, thus contributing to cancer development [7, 9–11]. Furthermore, a small number of studies implied that FBXO31 functioned as an oncoprotein to promote oncogenesis and cancer progression in lung and esophageal cancer [12–14]. Therefore, the opposite role of FBXO31 in different human cancers was critically associated with its downstream substrates, acting as oncogenic or tumor suppressive role. For example, identified oncogenic substrates of FBXO31 include MDM2, DUSP6, CyclinD1 and CyclinA1 [9, 11, 15, 16]. However, the physiological function of FBXO31 and its targeted substrates in PC have not been fully elucidated.

SIRT2 belongs to family members of NAD^+^-dependent type III protein deacetylase, which is predominantly localized in the cytoplasm [17]. SIRT2 is associated with histone deacetylation (H4K16Ac) and chromosomal condensation during mitosis [18]. Extensive research has been demonstrated that SIRT2 deacetylated APC/C activator CDH1 and CDC20 to activate APC/C, leading to Aurora-A degradation and mitosis regulation and genome integrity [19]. Studies have suggested that SIRT2 may be a novel target for cancer treatment [18, 20]. Oncogenic characteristic of SIRT2 was demonstrated in some types of cancer [21–23], while others showed the tumor suppressive role of SIRT2 [24–28]. Even in pancreatic cancer, the precise role of SIRT2 remains elusive and opposite [24, 29, 30]. Thus, the role and molecular mechanism of SIRT2 in PC need be better understood.

N^6^-methyladenosine (m6A) modification is one of the most abundant posttranscriptional modifications (PTMs) in eukaryotic mRNA [31, 32]. The well-known function of m6A is identified in regulating RNA metabolism, including pre-mRNA splicing, mRNA decay, mRNA translation, mRNA export [31, 32]. Growing body of evidence suggests that m6A modification is critically involved in tumorgenisis [33, 34]. In addition, m6A methylation is performed by m6A writer complex, which comprises a METTL3-METTL14 methyltransferase core and other components [31]. METTL3 serves as a catalytic subunit and METTL14 functions as a structural basis for substrate recognition [31]. It has been reported that METTL3 and METTL14 promote the tumor initiation and progression in PC [35–39]. Here, we identified a novel molecular mechanism of METTL3-FBXO31-SIRT2 signaling axis and its impact on tumor growth and metastasis in PC.

## Results

### FBXO31 is aberrantly up-regulated in PC tissues and associated with poor survival in PC patients

**Fig. 1:**
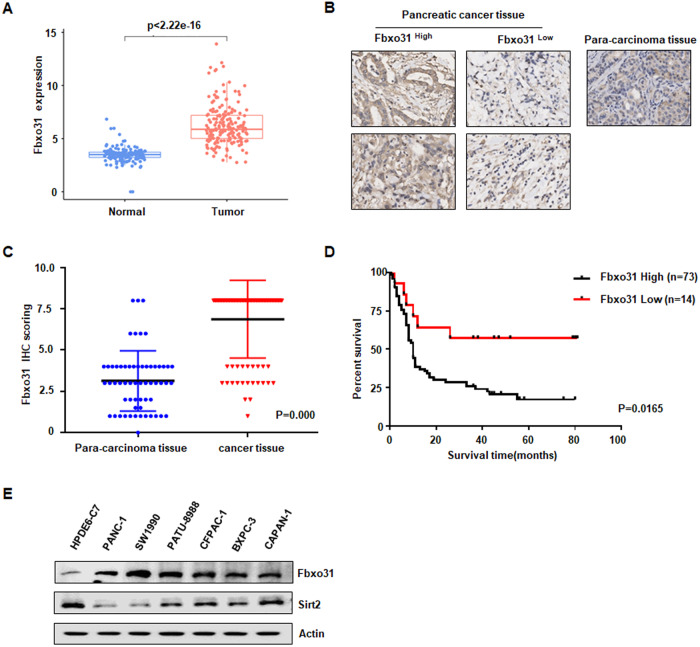
FBXO31 is up-regulated in pancreatic cancer tissues and cells. **A** FBXO31 mRNA expression in tumor and normal tissues from TGCA+GTEx cohort. **B** Representative images of FBXO31 IHC staining of human pancreatic cancer microarray slides. **C** IHC scores of PC tissues and adjacent normal tissues derived from human pancreatic cancer microarray slides for FBXO31 IHC staining. **D** Kaplan–Meier analysis of overall survival of PC patients based on FBXO31 expression (*n* = 87). **E** Western blotting analysis of FBXO31 and SIRT2 expression in different PC cells and HPDE6-C7 normal pancreatic epithelial cells.

To investigated the expression profile of FBXO31 in pancreatic adenocarcinoma (PAAD), which constitutes 90–95% of PC, we analyzed TCGA and GTEx clinical datasets, including 179 cases of PAAD tissues and 171 cases of adjacent normal tissues (167 cases from GTEx and 4 cases from TCGA). We found that FBXO31 mRNA expression levels were highly elevated in PAAD tissues compared to that in adjacent normal tissues (Fig. 1A). Furthermore, we measured FBXO31 protein expression by IHC staining in tissue microarray (TMA) containing 90 cases with PAAD and 60 adjacent normal tissues (Fig. 1B). IHC data of tissue microarray showed that cytoplasmic FBXO31 expression levels were up-regulated in tumor tissues compared to their adjacent normal tissues (Fig. 1C, *p* < 0.001). We also analyzed the overall survival of PAAD patients from tissue microarray by Kaplan–Meier analysis. The results showed that the patients with high FBXO31 expression had a shorter survival time than those with low expression of FBXO31 (Fig. 1D, *p* = 0.0165). Moreover, FBXO31 protein expressions were detected by Western blotting between human normal HPDE6-C7 pancreatic epithelial cell line and human PC cell lines, including Panc-1, PaTu-8988, SW1990, CFPAC-1, BxPC-3 and Capan-1 cells. The results exhibited that FBXO31 expression was higher in PC cell lines than in normal cell line (Fig. 1E). Taken together, it suggested that dysregulation of FBXO31 may be contributed to PC progression.

### FBXO31 promotes viability and motility of PC cells in vitro and in vivo

**Fig. 2:**
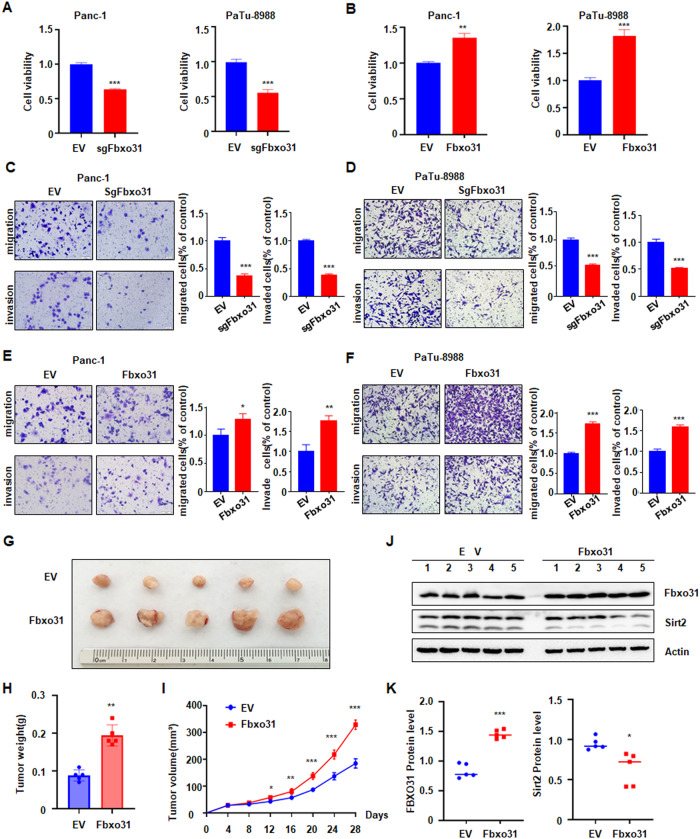
FBXO31 promotes PC development in vitro and in vivo. **A**, **B** MTT assays to detect cell growth of Panc-1 and Patu-8988 cells transfected with indicated FBXO31 sgRNA (**A**) or FBXO31 cDNA (**B**). Data are shown as mean ± SD of three independent experiments. **p < 0.01 compared to control, ***p < 0.001 compared to control. **C**, **D** Transwell assays to analyze cell migration and invasion capacity of Panc-1 cells (**C**) and Patu-8988 cells (**D**) transfected with FBXO31 sgRNAs. ***p < 0.001 compared to control. **E**, **F** Transwell assays to analyze cell migration and invasion capacity of Panc-1 cells (**E**) and Patu-8988 cells (**F**) transfected with FBXO31 cDNA. *p < 0.05, **p < 0.01, ***p < 0.001 compared to control. **G** Pictures of tumor mass dissected from FBXO31-overexpressing xenografts mouse models. Stable FBXO31 overexpression of Panc-1 cells and the control cells were injected subcutaneously into the BALB/c-nu/nu mice to establish xenografts mouse models. **H**, **I** Tumor weights (**H**) and tumor volumes (**I**) of dissected tumor mass in (**G**). **J**, **K** IB analysis of the FBXO31 and SIRT2 protein levels in the dissected tumors (**J**). The protein abundance of FBXO31 and SIRT2 was quantified and plotted (**K**).

To clarify the role of FBXO31 in PC, Patu-8988 and Panc-1 cells were transfected with FBXO31 cDNA for overexpression, and FBXO31 sgRNA for leading to FBXO31 knockdown, respectively. The mRNA expression levels of FBXO31 were verified using qRT-PCR in PC cells with FBXO31 overexpression and knockdown treatments (Fig. S1A). Moreover, the results of MTT assay showed that FBXO31 knockdown obviously inhibited cell viability in PC cells. (Fig. 2A). Consistently, ectopic expression of FBXO31 significantly enhanced cell viability in PC cells (Fig. 2B).

For measuring cell migratory ability, wound healing assay and Transwell migration assay were both performed in PC cells after FBXO31 modulation. The results of wound healing assay exhibited that FBXO31 deletion led to wound healing retardation compared to the control group (Fig. S1B). The wound scratch closure sped up in the group of FBXO31 overexpression compared to the control group in PC cells (Fig. S1C). Consistently, FBXO31 downregulation resulted in decreased migrated cells from the chamber, while FBXO31 upregulation led to increased migrated cells from the chamber (Fig. 2C–F). For Transwell invasion analysis, it was also proved that increased FBXO31 expression remarkably enhanced cell invasion capacity and decreased FBXO31 expression prominently suppressed cell invasion ability in PC cells (Fig. 2C–F). To determine whether FBXO31 promotes tumor growth in vivo, we subcutaneously injected Panc-1 cells with or without FBXO31 overexpression into the flank of nude mice. We found that FBXO31 overexpression enhanced tumor mass size, weights and volumes (Fig. 2G–I). Western blotting data showed the high expression of FBXO31 in tumor tissues (Fig. 2J, K). All of results showed that FBXO31 plays the oncogenic role in tumorigenesis and development in PC.

### SIRT2 is a direct target of FBXO31 in PC

**Fig. 3:**
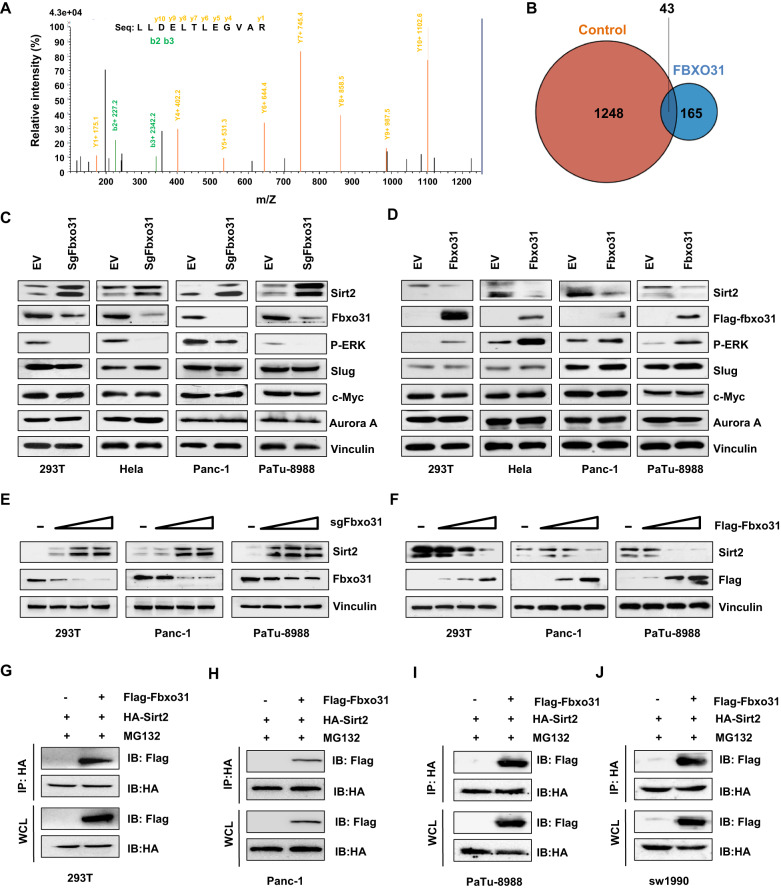
SIRT2 is a direct target of FBXO31 in PC. **A** Peptide sequence of SIRT2 interacting with Flag-FBXO31 identified by mass spectrometry. SIRT2 was one candidate protein. **B** Venn diagram analysis of candidate proteins derived from a co-IP-based mass spectrometry data in 293 T cells transfected with Flag-FBXO31 and EV. **C**, **D** IB analysis of WCLs derived from different cells transfected with FBXO31 sgRNA (**C**) and FBXO31 cDNA (**D**). **E**, **F** IB analysis of WCLs derived from different cells transfected with increased amounts of FBXO31 sgRNA (**E**) and FBXO31 cDNA (**F**). **G**, **J** IB analysis of immunoprecipitates (IPs) and WCLs derived from 293 T cells (**G**), Panc-1 cells (**H**), PaTu-8988 cells (**I**), and SW1990 cells (**J**) transfected with indicated plasmids. Cells were treated with 10 μM MG132 for 6 h before harvesting.

We therefore sought to explore the mechanisms of carcinogenic role of FBXO31 in PC. To identify novel targets of FBXO31, we screened for proteins combining with Flag-FBXO31 using a co-IP-based LC-MS/MS method in 293 T cells (Fig. 3A, B). We screened for common interacting proteins in the control and FBXO31 group through Venn diagram analysis (Fig. 3B and Supplementary File 1). Accordingly, SIRT2 was identified as one of FBXO31-associated protein (Fig. 3A). To this end, we found that SIRT2 protein abundance detected by western blotting was significantly increased after FBXO31 sgRNA transfection in different cell lines (Fig. 3C). Consistently, the protein abundance of SIRT2 was markedly reduced by FBXO31 cDNA transfection in a few of cell lines (Fig. 3D). Furthermore, FBXO31-deletion increased SIRT2 protein abundance in a dose-dependent manner (Fig. 3E), and similar results were also observed in FBXO31 overexpression group (Fig. 3F). We further elucidated the physical interaction between FBXO31 and SIRT2 using immunoprecipitation assay. We showed that FBXO31 specifically interacted with SIRT2 in HEK293T cells and different PC cell lines (Figs. 3G–J and S2). All of results demonstrated that SIRT2 is a specific target of FBXO31.

### FBXO31 promotes SIRT2 ubiquitination and degradation

**Fig. 4:**
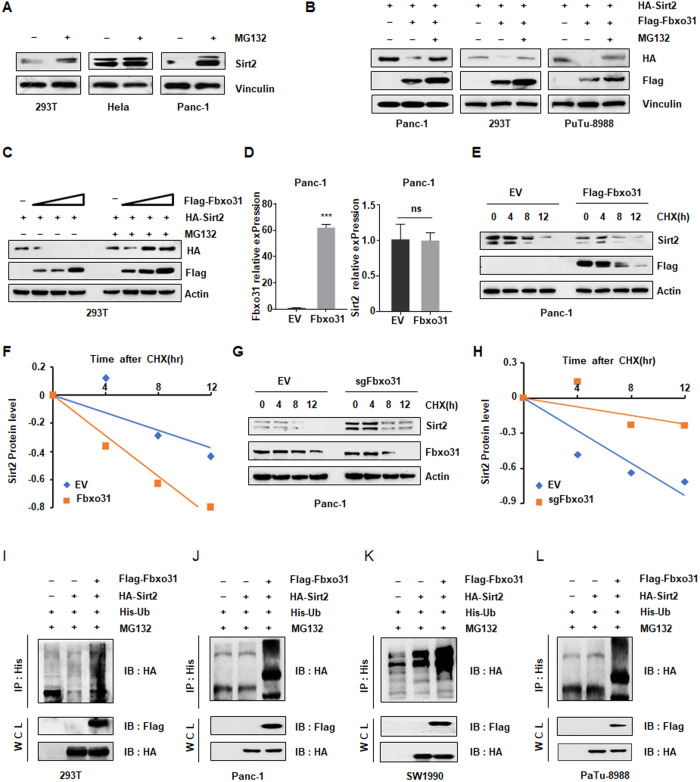
FBXO31 promotes SIRT2 ubiquitination and degradation. **A** IB analysis of whole cell lysates (WCLs) derived from 293 T cells, Hela cells and Panc-1 cells. Where indicated, cells were treated with 10 μM MG132 for 6 h before harvesting. **B** IB analysis of whole cell lysates (WCLs) derived from 293 T cells, PaTu-8988 cells and Panc-1 cells transfected with indicated plasmids, which treated with 10 μM MG132 for 6 h before harvesting. **C** IB analysis of whole cell lysates (WCLs) derived from 293 T cells transfected with SIRT2 cDNA and increased amounts of FBXO31 cDNA with or without 10 μM MG132 treatment. **D** qPCR analysis to detect FBXO31 and SIRT2 mRNA levels after FBXO31 overexpression in Panc-1 cells. Data are shown as mean ± SD of three independent experiments. ***p < 0.001 compared to EV. Ns nonsense. **E** IB analysis of WCLs derived from Panc-1 cells transfected with FBXO31 constructs. Where indicated, 100 μg/ml cycloheximide (CHX) was added and cells were harvested and lysed at indicated time points. **F** SIRT2 protein abundance in (**E**) was quantified by Image J and plotted. **G** IB analysis of WCLs derived from Panc-1 cells transfected with FBXO31 sgRNA. Where indicated, 100 μg/ml cycloheximide (CHX) was added and cells were harvested and lysed at indicated time points. **H** SIRT2 protein abundance in (**G**) was quantified by Image J and plotted. **I**–**L** IB analysis of products of ubiquitination and WCLs derived from 293 T cells (**I**), PaTu-8988 cells (**J**), Sw1990 cells (**K**) and Panc-1 cells (**L**) transfected with indicated constructs. Cells were treated with 10 μM MG132 for 6 h before harvesting.

As FBXO31 serves as substrate recognition subunit of E3 ubiquitin ligase [5, 11], we speculated whether FBXO31 negatively regulated SIRT2 protein abundance through proteasome-dependent degradation. To this end, we found that the endogenous SIRT2 protein expression was prominently increased upon treating HEK293T, HeLa and Panc-1 cells with proteasome inhibitor MG132 (Fig. 4A). Furthermore, FBXO31-mediated destruction of SIRT2 could be blocked by MG132 in several cell lines, which were pretreated with MG132 and co-transfection of FBXO31 and SIRT2 (Fig. 4B, C). Impressively, FBXO31 hardly changed the mRNA expression level of SIRT2 in multiple cell lines treated with either FBXO31 overexpression or FBXO31 knockdown (Figs. 4D and S3A–C). Moreover, cycloheximide experiments showed the half-life time of endogenous SIRT2 protein was shortened after FBXO31 ectopic expression (Figs. 4E, F and S3D, E). Consistently with these findings, half-life of SIRT2 protein was lengthened after FBXO31 deletion (Figs. 4G–H and S3F–G), suggesting that FBXO31 regulated SIRT2 expression in a PTM manner. Considering the above-mentioned results, which declared that FBXO31 controlled SIRT2 expression and bound to SIRT2, we next detected whether FBXO31-mediated destruction of SIRT2 is a consequence of FBXO31-catalyzed ubiquitination. The results showed that ectopic expression of FBXO31 remarkably increased the amount of SIRT2 ubiquitination (Fig. 4I–L). Taken together, these results suggested that FBXO31 was the physiological E3 ligase that interacted with SIRT2 and promoted Sir2 ubiquitination and degradation in PC.

### FBXO31-mediated destruction of SIRT2 is dependent on the F-box motif of FBXO31 and the Sirtuin-type motif of SIRT2

**Fig. 5:**
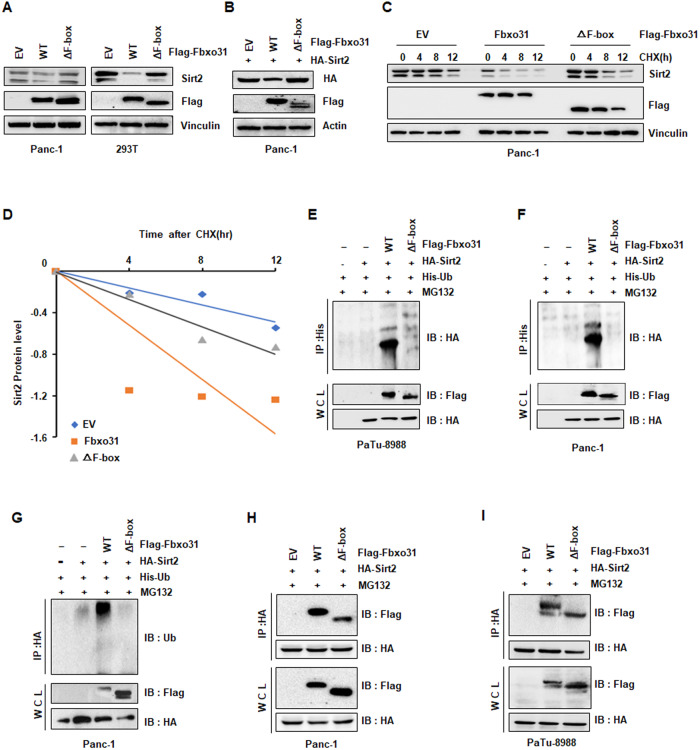
FBXO31 mediates destruction of SIRT2 through its F-box motif, but not for substrate binding. **A** IB analysis of WCLs derived from 293 T cells and Panc-1 cells transfected with indicated plasmids. **B** IB analysis of WCLs derived from Panc-1 cells transfected with indicated plasmids. **C** IB analysis of WCLs derived from Panc-1 cells transfected with indicated constructs. Where indicated, 100 μg/ml cycloheximide (CHX) was added and cells were harvested and lysed at indicated time points. **D** SIRT2 protein abundance in (**C**) was quantified and plotted. **E**, **G** IB analysis of products of ubiquitination and WCLs derived from PaTu-8988 cells (**E**), Sw1990 cells (**F**), and Panc-1 cells (**G**) transfected with indicated constructs. Cells were treated with 10 μM MG132 for 6 h before harvesting. **H**, **I** IB analysis of IPs and WCLs derived from Panc-1 cells (**H**) and PaTu-8988 cells (I) transfected with indicated plasmids. Cells were treated with 10 μM MG132 for 6 h before harvesting.

It has been reported that F-box domain of FBXO31 was required for substrate ubiquitination and degradation [7, 11], but not for substrate binding [40]. It has suggested that FBXO31 interacted with SCF through its F-box domain [40]. To determine whether FBXO31 mediated the degradation of SIRT2 through its F-box motif, we constructed FBXO31 ΔF-box-mutant plasmid. As shown in western blotting assays, FBXO31 ΔF-box mutation did not decrease the protein abundance of SIRT2 in contrast to wild-type (WT) FBXO31 (Figs. 5A, B and S4A). Furthermore, we observed that FBXO31 WT, but not FBXO31 ΔF-box, essentially decreased the half-life of SIRT2 in Panc-1 cells and 293 T cells (Figs. 5C, D and S4B, C). Consistently, ectopic expression of wild-type FBXO31, but not FBXO31 ΔF-box, enhanced SIRT2 ubiquitination (Fig. 5E–G). All above results suggested that FBXO31-mediated degradation of SIRT2 is dependent on its F-box domain. To test whether F-box domain of FBXO31 is necessary for binding to SIRT2, we further performed Co-IP experiments. The results showed that both FBXO31 WT and ΔF-box mutant FBXO31 could interact with SIRT2 (Fig. 5H–I), suggesting that F-box domain was not required for substrate recognition of FBXO31.

**Fig. 6:**
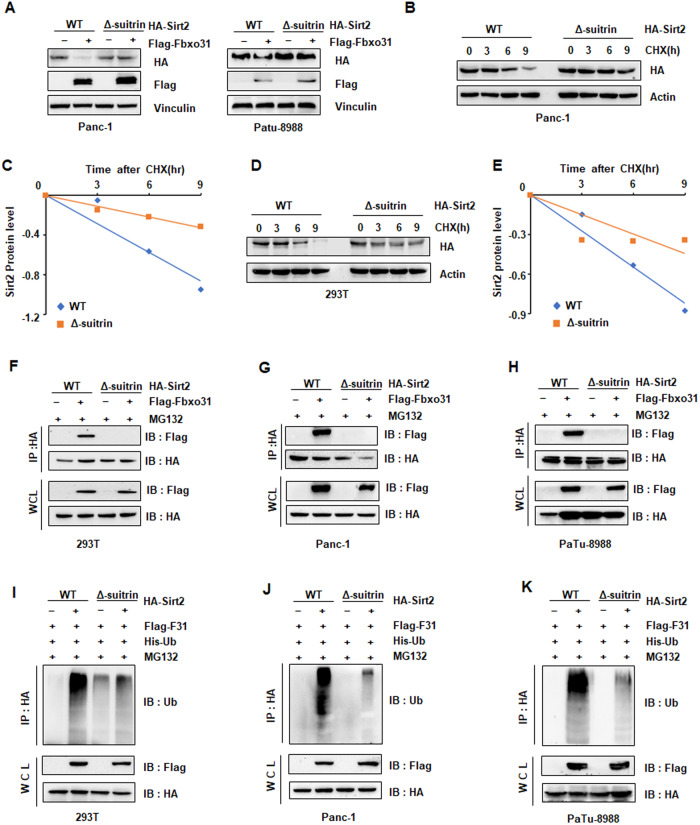
Sirtuin domain of SIRT2 was involved in FBXO31 binding and ubiquitination. **A** IB analysis of WCLs derived from Panc-1 cells and PaTu-8988 cells transfected with indicated plasmids. **B**, **C** IB analysis of WCLs derived from Panc-1 cells transfected with indicated constructs (**B**). Where indicated, 100 μg/ml cycloheximide (CHX) was added and cells were harvested and lysed at indicated time points. SIRT2 protein abundance in (**B**) was quantified and plotted as indicated (**C**). **D**, **E** IB analysis of WCLs derived from 293 T cells transfected with indicated constructs (**D**). Where indicated, 100 μg/ml cycloheximide (CHX) was added and cells were harvested and lysed at indicated time points. SIRT2 protein abundance in (**D**) was quantified and plotted as indicated (**E**). **F**, **H** IB analysis of IPs and WCLs derived from293T cells (**F**), Panc-1 cells (**G**), and PaTu-8988 cells (**H**) transfected with indicated plasmids. Cells were treated with 10 μM MG132 for 6 h before harvesting. **I**–**K** IB analysis of products of ubiquitination and WCLs derived from 293T cells (**I**), Panc-1 cells (**J**), and PaTu-8988 cells (**K**) transfected with indicated plasmids. Cells were treated with 10 μM MG132 for 6 h before harvesting.

As noted above, it has shown that FBXO31 acts as an upstream E3 ligase of SIRT2, thereby, we considered the binding sites of SIRT2 to specifically interact with FBXO31. Referring to the domain information of SIRT2 derived from UniProt database (UniProtKB-Q8IXJ6), we generated a truncated SIRT2 mutant containing sirtuin -type domain deletion (named as SIRT2 Δ-sirtuin). We showed that SIRT2 Δ-sirtuin blocked FBXO31-mediated SIRT2 downregulation in Panc-1 and PaTu-8988 cells (Fig. 6A). And the half-life of SIRT2 Δ-sirtuin was lengthened compared with SIRT2 WT in Panc-1 and 293 T cells (Fig. 6B–E). In support of the sirtuin-type domain of SIRT2 contributing of a key role in FBXO31-mediated SIRT2 degradation, Co-IP results showed that SIRT2 WT, instead of SIRT2 Δ-sirtuin, interacted with FBXO31 (Fig. 6F–H). Consistently, ubiquitination of SIRT2 was significantly decreased in SIRT2 Δ-sirtuin, but not in SIRT2 WT (Fig. 6I–K). These results suggested sirtuin domain of sirt2 was involved in FBXO31 binding and its ubiquitination.

### SIRT2 is required for FBXO31-mediated promotion effect of cell viability, migration and invasion in PC

Owing to that the exact role of SIRT2 in PC remains controversial [24, 29, 30], we elucidated the biological functions of SIRT2 in PC in vitro and vivo. To this end, we analyzed cell viability, migration and invasion ability after PC cells treated with SIRT2 ectopic expression or deletion (Fig. S5A). We found that ectopic expression of SIRT2 significantly inhibited cell viability, while SIRT2 deletion obviously promoted cell viability in Panc-1 and PaTu-8988 cells (Fig. S5B, C). Furthermore, we also showed that cell migration and invasion capacities were remarkably elevated by SIRT2 overexpression, but were depressed by SIRT2 depletion in PC cells (Fig. S5D–I). Moreover, we demonstrated SIRT2 deletion in Panc-1 cells inhibited the growth of xenografted tumor in mice, revealing a significantly reduction in tumor weights and volumes (Fig. S6A–D).

**Fig. 7:**
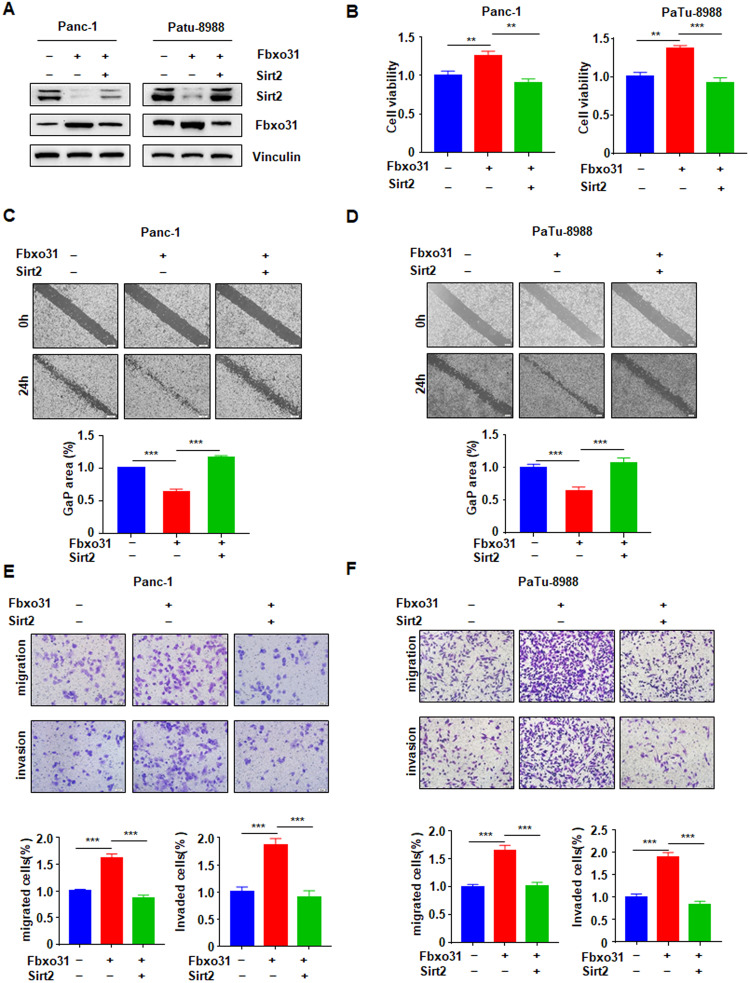
SIRT2 is required for FBXO31-mediated promotion of cell viability, migration and invasion in PC. **A** IB analysis of WCLs derived from Panc-1 cells and PaTu-8988 cells transfected with indicated plasmids. **B** MTT assays to detect cell proliferation of Panc-1 and Patu-8988 cells transfected with indicated plasmids. Data are shown as mean ± SD of three independent experiments. **p < 0.01 and ***p < 0.001. **C**, **D** Wound healing assays to detect cell migration of Panc-1 cells (**C**) and PaTu-8988 cells (**D**) transfected with indicated plasmids. ***p < 0.001. **E**, **F** Transwell assays to detect cell migration and invasion of Panc-1 cells (**E**) and PaTu-8988 cells (**F**) transfected with indicated plasmids. ***p < 0.001.

To understand the role of FBXO31 via targeting SIRT2 degradation, we performed rescue experiments through simultaneous overexpression or deletion of FBXO31 and SIRT2 on cell viability and cell mobility. PC cells were cotransfected with FBXO31 and SIRT2, or with sgFBXO31 and shSIRT2. The protein abundances of FBXO31 and SIRT2 were detected by western blotting to verify simultaneous overexpression or deletion (Figs. 7A and S7A). Moreover, overexpression of SIRT2 weakened the promotion effect of FBXO31 overexpression on diverse oncogenic phenotypes, including cell growth (Fig. 7B), cell migration (Fig. 7C–F) and cell invasion (Fig. 7E, F). In agreement with these findings, deletion of SIRT2 enhanced the inhibitory effect of FBXO31 deletion on cell viability, cell migration and cell invasiveness (Fig. S7B–E).

Taken together, our results showed that FBXO31 promoted PC cell viability and motility partly through mediating SIRT2 degradation. In addition, we detected the express of several downstream targets of SIRT2, including c-Myc [29], Aurora A [19], Slug [41], p-ERK [24], after cells were treated with FBXO31 ectopic expression and knockdown in multiple cells. We found only p-ERK expression, rather than c-Myc, Aurora A and Slug, was down-regulated by FBXO31 knockdown and up-regulated by FBXO31 overexpression (Fig. 3C, D). Moreover, we found that sh-Sirt2 transfection induced cell viability, migration and invasion in Panc-1 and PaTu-8988 cells, which were abrogated by ERK inhibitor treatment (Fig. S8A-F). These findings suggest that SIRT2 performed its functions in part via the ERK pathway. The results may suggest that FBXO31-mediated SIRT2 degradation and subsequently leading to p-ERK downregulation are responsible for the oncogenic function of FBXO31 in PC.

### METTL3/METTL14 induces FBXO31 m6A modification and promotes FBXO31 mRNA translation

**Fig. 8:**
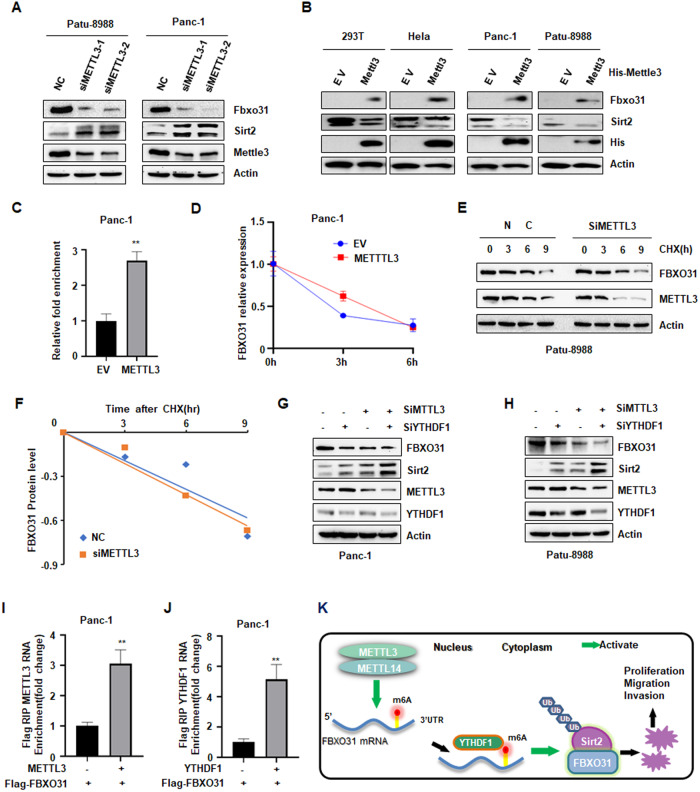
METTL3 induces FBXO31 m6A modification and promotes FBXO31 mRNA translation. **A** IB analysis of WCLs derived from Panc-1 cells and PaTu-8988 cells transfected with METTL3 siRNAs. **B** IB analysis of WCLs derived from a number of cells transfected with METTL3 cDNA. **C** Enrichment of mRNA by A/G magnetic beads coupled with m6A antibody in Panc-1 cells, m^6^A-IP combined with RT-qPCR was used to quantify the relative m6A level of Fbxo31 mRNA. **D** qPCR analysis of FBXO31 mRNA levels in Panc-1 cells in the absence or presence of METTL3 overexpression, and after actinomycin D treatment. **E**, **F** IB analysis of WCLs derived from PaTu-8988 cells transfected with indicated constructs (**E**). Where indicated, 100 μg/ml cycloheximide (CHX) was added and cells were harvested and lysed at indicated time points. FBXO31 protein abundance in (**E**) was quantified and plotted as indicated (**F**). **G**, **H** IB analysis of WCLs derived from Panc-1 cells (**G**) and PaTu-8988 cells (**H**) transfected with indicated siRNAs. **I**, **J** The interaction between FBXO31 mRNA and METTL3 (**I**) or YTHDF1 (**J**) was analyzed by RIP from Panc-1 cells immunoprecipitated with Flag antibody. **K** The diagram of FBXO31 increased by METTL3 to promote PC development via regulating SIRT2 ubiquitination and degradation.

It has been reported that METTL3 and METTL14 serve as oncogenes in PC [35–37]. In agreement with other reports, we also observed that METTL3 could enhance cell viability and cell invasion in PC cells (Fig. S9A–D). However, it is poorly understood whether FBXO31 mRNA could be modified by m6A readers, such as METTL3 or METTL14. To this end, we exhibited that METTL3 deletion contributed to obviously decreased protein expression of FBXO31 and increased protein expression of SIRT2 in PC cells (Fig. 8A). However, overexpression of METTL3 significantly increased FBXO31 protein abundance and subsequently decreased SIRT2 protein abundance (Fig. 8B). As known as an adaptor of METTL3 for RNA binding and allosteric activator of the enzymatic activity of METTL3 [31], we further investigated whether METTL14 was involved in regulating FBXO31 expression. Similar results showed that METTL14 also positively regulated FBXO31 protein expression and negatively regulated SIRT2 protein expression (Fig. S10A, B). Furthermore, our meRIP-qPCR results showed that METTL3 could methylate FBXO31 mRNA (Fig. 8C). However, qRT-PCR results showed that METTL3 had no significantly influence over FBXO31 mRNA level (Figs. S9E–G). We also demonstrated METTL14 did not altered FBXO31 mRNA level (Fig. S10C, D). Moreover, mRNA stability assay by actinomycin D showed that METTL3 didn’t change FBXO31 mRNA half-life (Fig. 8D). Taken together, these results suggested that METTL3 or METTL14-mediatied upregulation of FBXO31 might be involved in translational control rather than mRNA decay regulation.

In addition, we performed cycloheximide assay to exclude whether METTL3 might influence FBXO31 protein stability. The results exhibited that FBXO31 protein elimination has no significantly change between METTL3 overexpression and the control group (Fig. 8E, F). As we known that m6A signals are recognized by m6A-binding proteins, such as YTHDF1, YTHDF2, or YTHDF3 [31]. We therefore explored whether YTHDF-1 was involved in METTL3-mediated translation regulation. Firstly, we performed western blotting to detect FBXO31 expression in cells treated with YTHDF-1 deletion and METTL3 overexpression. The results showed that YTHDF1 deletion robustly reduced METTL3-mediated enhanced translation of FBXO31 (Fig. 8G, H). Moreover, RIP-qPCR assay showed that METTL3, YTHDF1 directly bound to FBXO31 mRNA (Fig. 8I, J). Thus, our works suggested that METTL3 upregulated FBXO31 due to enhancing translation rather than decreasing RNA decay and protein stability, and METTL3 promoted FBXO31 translation in an YTHDF1-dependent manner.

## Discussion

In recent decades, a variety of therapeutic strategies have been developed and substantially improvements have been received in the patient survival of some cancers. Nevertheless, to date, the treatment of PC remains a terrible challenge and has proved refractory to these new therapeutic approaches. Thus, the demand for understanding the complex molecular mechanisms and epigenetic alterations of PC is high. In the present study, we demonstrate that METTL3/METTL14 enhanced m6A modification of FBXO31 mRNA and upregulated FBXO31 protein expression, subsequently leading to SIRT2 ubiquitination and degradation (Fig. 8K). We identify a novel METTL3/METTL14-FBXO31-SIRT2 signaling axis in the regulation of PC progression, which contributes to provide new and promising targets of PC treatment.

Extensive research has been indicated that E3 ligases play a critical role in tumor development and serve as therapeutic targets for tumor treatment [6, 42]. FBXO31 has been frequently suggested to function as tumor suppressor or oncogenic gene in different tumors [8–10, 13, 14]. However, the function and its molecular mechanism of FBXO31 in PC are fuzzy. Herein, we detected biological function of FBXO31 through IHC staining for human PC samples, in vivo and in vitro experiments. Our results showed that FBXO31 was up-regulated and associated with poor survival in PC samples. FBXO31 promoted cell viability and motility in vitro and enhanced tumor growth in vivo. Hence, we provide abundant proof to demonstrate an oncogenic role of FBXO31 in PC progression, suggesting FBXO31 may be a potential therapy target for PC.

As an E3 ligase, FBXO31 targets different substrates for ubiquitination and destruction. For underlying mechanism analysis, we identified SIRT2 as a novel substrate of FBXO31 in PC. We found that FBXO31 decreased SIRT2 protein abundance, not mRNA level. Moreover, half-life time of SIRT2 was shortened by FBXO31 overexpression through Cycloheximide analysis. FBXO31 could bind to SIRT2 and strengthen SIRT2 ubiquitination and subsequent degradation. Consistent with previously report [40], we identified that F-box domain of FBXO31 was required for FBXO31-mediated SIRT2 ubiquitination and degradation, but not for substrates recognition of FBXO31. Furthermore, we suggested that FBXO31 binds to sirtuin domain of SIRT2 and strengthens SIRT2 ubiquitination and subsequently degradation. Thus, we identified a novel substrate of FBXO31 and FBXO31 acts as an upstream E3 ligase to mediate SIRT2 degradation in a posttranslational manner.

The opposite roles of SIRT2 in PC were observed in previously reports [29]. It has been reported that c-Myc increased SIRT2 expression in MiaPaca-2 PC cells, and SIRT2 promoted c-Myc and N-Myc stabilization, leading to cell growth promotion [29]. Consistent with this finding, other study showed that SIRT2 deacetylated LDH-A K5 and enhanced LDH-A enzyme activity and protein level, which caused significant increase of cell viability and cell migration in PC [30]. However, another report supported SIRT2 deficiency increased KrasG12D mice tumorigenic transformation, including development of PanIN and progression to PDAC [24]. SIRT2 loss enhanced KRAS acetylation and activity, leading to increased p-ERK expression [24]. In this study, we demonstrated that SIRT2 plays tumor suppressive role in PC. SIRT2 inhibited cell proliferation and cell invasion in Panc-1 and PaTu-8988 cells in vitro. In addition, SIRT2 deficiency in Panc-1 cells accelerated xenografts tumor growth in vivo. More importantly, rescue experiments exhibited that SIRT2 is involved in FBXO31-mediated proliferation and motility of PC cell. Consistently, we found that FBXO31 mediated p-ERK protein expression instead of other SIRT2 downstream targets, including c-Myc and Aurora. These different conclusions regulating the role of SIRT2 in PC could be due to using various cell lines and animal models, which is required to further use Sirt2 conditional transgenic mouse models and Sirt2 conditional knockout mouse models to fully elucidate the function of SIRT2 in PC.

Extensive research revealed that m6A levels were elevated in PC and higher m6A levels were correlated with poor survival of PC patients [35, 37]. Importantly, METTL3 had higher expression in PC tissues compared with adjacent-normal tissues [35]. Upregulation of METTL3 promoted PC cell growth and motility [35], and enhances chemo- and radio-resistance in PC cells [36]. All of results suggested that m6A and METTL3 play oncogenic roles in PC. Similar results were observed in studies of the function of METTL14 in PC [37–39], which suggested that METTL14 contributes to oncogenic role in PC as well. Whether FBXO31 is regulated via epigenetic modification remains unclear. In this study, we found oncogenic METTL3 or METTL14 elevated FBXO31 protein expression and declined SIRT2 protein expression. These results disclosed that FBXO31 may be m6A-modified by METTL3/METTL14. It is becoming more evident that METTL3 owes methyltransferase activity and METTL14 possesses RNA-binding sites and functions as allosteric activator of METTL3 [31]. Thus, we pursue how METTL3, the chief of m6A writer, regulates FBXO31 m6A modification. RT-PCR and mRNA stability assay exhibited that the expression of FBXO31 mediated by METTL3 was not due to METTL3-induced mRNA decay regulation. Furthermore, cycloheximide assay exclude the probability of METTL3-induced FBXO31 protein stability. Moreover, meRIP, RIP and western blotting assay demonstrated that METTL3 induced FBXO31 m6A modification through enhancing FBXO31 translation in an YTHDF1-dependent manner. Taken together, we identify an oncogenic role of FBXO31 in PC, as well as clarify its upstream epigenetics regulation and its novel downstream substrate. Our study offers novel insights into molecular basis of METTL3-FBXO31-SIRT2 axis and provides the opportunity to the development of potential therapeutic strategies for PC.

## Materials and methods

### Human pancreatic cancer tissue and IHC

Human pancreatic cancer microarray slides (HpanA150su01) were purchased from OUTDO BIOTECH (Shanghai, China). The tissue chip contains 90 cases of pancreatic cancer tissues and 60 cases of matched tumor-adjacent normal tissues. IHC stain was operated to detect the expression of FBXO31 and SIRT2 in the same tissue chips as described before [43]. Slides were treated by deparaffinization and rehydration operation. Then, slides were pretreated with antigen retrieval, and incubated with 3% H_2_O_2_ for 10 min at room temperature (RT). After blocking with BSA for 1 h, slides were incubated with human FBXO31 or SIRT2 antibodies overnight at 4 °C. Slides were incubated with biotinylated secondary Ab for 1 h at RT, followed by streptavidin-conjugated horseradish peroxide for 1 h and DAB for 5 min at RT. Images were collected by Aperio Imagescope software and analyzed by two independent pathologists. IHC staining was scored as follows: score = percentage of positive staining of tumor cells (0: 0%; 1: 1–25%; 2: 26–50%; 3: 51–75%; 4: 76–100%) × staining intensity (0: negative; 1: weak; 2: medium; 3: strong). The final scores ≥4 were considered as high expression of FBXO31 or SIRT2 for statistical analysis.

### Cell culture, cell transfection and infection

HEK293T, HeLa and human pancreatic cancer cells (Patu-8988 and Panc-1) were obtained from the Cell Bank of the Chinese Academy of Sciences (Shanghai, China). The cells were authenticated by STR profiling and tested for mycoplasma contamination. All cells were maintained in DMEM medium supplemented with 10% FBS, and 1% Penicillin/Streptomycin. Cells were cultured at 37 °C at 5% CO_2_ atmosphere. Plasmid constructs or siRNAs were transfected using Lipofectamine 3000 according to manufacturer’s protocol. Lentiviral vectors loading FBXO31 cDNA or SIRT2 shRNA sequences were packaged and infected PC cells for generation of stable transfected cells.

### Plasmids, siRNA and sgRNA

Plasmids, including FBXO31, FBXO31-Δ-Fbox, SIRT2, Del-SIRT2, METTL3 and METTL14, were obtained from Youbio Biological Technology (Hunan, China). They were constructed into pcDNA 3.1 vector carrying with different tags. FBXO31 sgRNAs were designed using guide design tools from Zhang lab (https://zlab.Bio/guide-design-resources) and cloned into CRISPR V2 backbones. FBXO31-sgRNA sequence was as follows: CAG GCT TGA TGA GGT CGT CGG GG. SIRT2 shRNA sequence was cloned into lentiviral vector pHBLV-U6 (Hanbio Bio, Shanghai, China) indicated as follows: GCC AAC CAT CTG TCA CTA CTT. The siRNAs of METTL3, METTL14, YTHDF1 and YTHDF2 were purchased from Hanbio Biotechnology Co. LTD (Shanghai, China). METTL3 siRNA sequences were used as described before [44]: siMETTL3-1, CTG CAA GTA TGT TCA CTA TGA; siMETTL3-2, GCA AGT ATG TTC ACT ATG AAA. METTL14 siRNA sequences were used as indicated before [45]: SiMETTL14-1, GCT GGA CTT GGG ATG ATA TTA; siMETTL14-2, GAA CCT GAA ATT GGC AAT ATA. siRNA sequences of YTHDF1 and YTHDF2 were performed as described before [46, 47]. siYTHDF1-1, CCT CCA CCC ATA AAG CAT A; siYTHDF1-2, CCT GCT CTT CAG CGT CAA TTT-3; siYTHDF2, AAG GAC GTT CCC AAT AGC CAA.

### Antibodies

Anti-FBXO31 antibody (#86137; 1:1000) antibody was purchased from Abcam. Anti-AuroraA antibody (#14475; 1:1,000), anti-Slug antibody (#9585; 1:1000), anti-C-myc antibody (#5605; 1:1000), anti-p-p44/42 MAPK (Erk1/2) (#4370; 1:1000) and anti-tubulin antibody (2128 S; 1:2000) antibodies were all obtained from Cell Signaling Technology. Anti-SIRT2 (66410-1-Ig; 1:1500), anti-METTL3 (15073-1-AP; 1:1500), anti-METTL14 (26158-1-Ap; 1:1500), Anti-Flag tag (20543-1-AP; 1:1500), anti-HA tag (51064-2-AP; 1:1000), Anti-β-actin (20536-1-AP; 1:2000) and anti-His tag (66005-1-Ig; 1:1,000) antibodies were all bought from Proteintech. Peroxidase-conjugated anti-mouse secondary antibody (70-GAM007, 1:5000) and peroxidase-conjugated anti-rabbit secondary antibody (70-GAR0072; 1:5,000) were purchased from MultiSciences Company.

### MTT assay

Cell viability was conducted by MTT assay as described before [43]. PC cells pretreated with indicated groups were seeded into 96 well plates. After 72 h, 10 μl MTT solution (0.5 mg/ml) was added into each well of plates and cultured for 4 h. After discarding the supernatant of each well, 100 μl DMSO was added. Then, OD490 value was detected and recorded.

### Wound healing assay

Wound healing assay was performed to detect cell migration as described before [43]. Transfected cells with indicated groups were incubated in 6-well plates. After cell confluence reached over 90%, a linear wound scratch was made by scraping the surface of plates using a pipette tip. Then, the wound scratches at 0 h and 20 h were photographed, respectively.

### Transwell migration and invasion assay

Transwell assay was performed as mentioned previously to analyze cell migration and invasion ability [43]. Transfected cells were seeded into Transwell upper-chamber with serum-free medium, while medium containing 10% FBS was placed into lower chamber (Corning). For Transwell invasion assay, the chamber should be precoated with a thin-layer Matrigel (BD Biosciences) before cells were seeded. Consequently, the upper cells of chambers were removed using a cotton buds, and the bottom surface cells of chambers were fixed, stained and photographed.

### qRT-PCR

Total RNA was extracted using TRIzol (Ambion) and reverse transcribed into cDNA using PrimeScript™ RT reagent Kit (TaKaRa). Real-time PCR was carried out using SYBR Green PCR Master Mix and a LightCycler480 Instrument (Roche). The data were quantified by 2^−ΔΔCt^ method. Primers were described as follows: FBXO31, sense, CAT CAA GCC TGG CCT CTT C, antisense; GGT CGA TCT CCA CTG TCT G; SIRT2, sense, CAC ATC ACA CTG CGT CAG C, antisense, CTT CAC ACT TGG GCG TCA C; METTL3, sense, TTG TCT CCA ACC TTC CGT AGT, antisense, CCA GAT CAG AGA GGT GGT GTA G; METTL14, sense, AGT GCC GAC AGC ATT GGT G, antisense, GAG CAG AGG TAT CAT AGG AA; YTHDF1, sense, GCA CAC AAC CTC CAT CTT CG, antisense, AAC TGG TTC GCC CTC ATT GT; GAPDH, sense, CAG CCT CAA GAT CAG CA, antisense, TGT GGT CAT GAG TCC TTC CA.

### Western blotting

Cells were collected and lysed in cold RIPA buffer addition of protease inhibitor cocktail (Thermo Fisher Scientific). The lysates were centrifuged at 12,000 *g* for 20 min at 4 °C and then the liquid supernatants were collected. The protein concentrations were estimated by BCA methods. Then, the proteins were electrophoresed using SDS-PAGE gel and transferred to PVDF membrane. The members were probed with the indicated primary antibodies overnight at 4 °C. After washing with TBST buffer for 3 times, the members were incubated proper secondary antibodies for 2 h at RT. The signals were visualized using chemiluminescence reagents (Thermo Fisher Scientific).

### Immunoprecipitation

For Immunoprecipitation analysis, cells were lysed with cold NP40 lysate with protease inhibitor cocktail (Thermo Fisher Scientific). Then, the proteins were added and incubated with magnetic beads supplemented with HA or Flag Tag (Sino biological, China). After washing 3 times using TBST buffer, the beads were eluted by boiling in SDS loading buffer. Subsequently, the final elute was subjected to SDS-PAGE electrophoresis and western blotting analysis.

### In vivo ubiquitination assay

To detect in vivo ubiquitination of SIRT2, cells were transfected with His-ubiquitin along with indicated plasmids. After 36 h post-transfection, cells were incubated with MG132 (10 μM, Sigma) for 6 h. Then, the cells were collected and lysed in NP40 buffer. The lysates were cultured with HA-tag magnetic beads. The beads were washed for 3 times by TBST and eluted by boiling in SDS loading buffer. Then the pull-down protein was analyzed by western blotting.

### Tandem mass spectrometry

HEK293T cells transfected with Flag-FBXO31 or EV were immunoprecipitated with Flag magnetic beads described as Flag IP. The lysates were resolved on SDS-PAGE and stained by Coomassie blue. The band of FBXO31-associated protein was excised from the gel and sent for tandem mass spectrometric peptide analysis. The LC-MS/MS were performed at Shanghai Applied Protein Technology (China). The resulting raw data were processed using Mascot2.2 software to search specific peptide sequences from UniProt database.

### Protein stability analysis

Cycloheximide method was performed to analyze protein half-life for protein stability studies. Cells were treated with indicated transfection. Then, cells were cultured with 20 μg/ml cycloheximide (Sigma) after 48 h post-transfection. To detect protein abundances, the cells were lysed and proteins were measured by western blotting at indicated time points.

### mRNA stability analysis

Actinomycin D, an inhibitor of transcription, was used to detect RNA stability. The transfected cells were treated with 5 μg/ml actinomycin D (MCE, USA). Then, the cells were harvested at indicated time points and the total RNAs were extracted by TRIzol reagent. The relative expression of FBXO31 mRNA was analyzed by qRT-PCR. The half-life time of FBXO31 mRNA were calculated as described before [48].

### MeRIP-qPCR assay

MeRIP-PCR assay was performed as described before [49]. Total RNA was isolated from cells treated with indicated groups, and mRNA was isolated using NEBNext Poly(A) mRNA Magnetic Isolation Module (NEB). 5 μg Puried mRNA was fragmented to 100–200 nt by NEBNext® Magnesium RNA Fragmentation Module (NEB). Next, 10% of fragmented mRNA was saved as input solution and the rest of fragmented mRNA was immunoprecipitated with m6A antibody (Abcam, ab151230) in IP binding buffer (10 mM Tris-HCl, 150 mM NaCl, 0.1% NP-40, pH 7.5) for 2 h at 4 °C. Then, 50 μl washed protein A/G magnetic beads (Thermo) were added to the immunoprecipitation mixture for 2 h at 4 °C. After incubation, the beads were collected and washed 2 times by IP binding buffer, subsequently 2 times by low-salt-reaction buffer (50 mM NaCl, 10 mM Tris–HCl, pH 7.5, 0.1% NP-40), and 2 times using high-salt-reaction buffer (500 mM NaCl, 10 mM Tris–HCl, pH 7.5, 0.1% NP-40). The bound RNA was eluted and extracted with TRIzol, and qRT-PCR was performed.

### RIP-qPCR assay

Cells were transfected with indicated group. After 48 h, cells were collected and lysed in RIPA buffer containing protease inhibitor and RNase inhibitor. Cell lysate were collected after centrifugation at 12,000 *g* for 15 min, and 10% volume of lysate were used as input. Then, the rest of lysate were incubated with indicated antibody at 4 °C overnight followed by incubation with 50 μl protein A/G magnetic beads for 2 h at 4 °C. After washing the beads 3 times, RNA was extracted with TRIzol, and qRT-PCR was performed.

### Animal experiments

Xenografted implantation model was established to estimate the in vivo effect of FBXO31 and shSIRT2. Five-weeks-old female nude mice were employed for subcutaneous injections. Panc-1 cells with stable expression of FBXO31 or shSIRT2 and their control cells were injected into the flanks of nude mice. The xenografted tumors were measured using calipers every 5 days. The recipient mice were euthanized after 4 weeks of injections and the tumor masses were resected. All animal studies were approved by the Animal Experimentation Ethical Committee of Bengbu Medical University (Bengbu, Anhui, China).

### Statistical analysis

All data were statistically analyzed by using SPSS 21 and GraphPad Prism 9 and presented as mean ± SD. The significance was determined by two-tailed paired or unpaired t test for two groups, and ANOVA test for multiple groups. Chi‑squared test was performed to estimate the distributions of clinicopathological variables. The Kaplan–Meier method was used to analyze the overall survival of PC patients.

### Supplementary information

supplementary file

Supplementary file legends

Supplementary figures

Original WB Images

## Data Availability

The datasets supporting the conclusions of this article are included within the article and its additional files.
