# Supplementary material for: FBXO31 is upregulated by METTL3 to promote pancreatic cancer progression via regulating SIRT2 ubiquitination and degradation
Source: Cell Death Dis. 2024 Jan 12;15(1):37. doi: 10.1038/s41419-024-06425-y (PMC10786907; doi:10.1038/s41419-024-06425-y)
Supplement: Supplementary file 2 — Supplementary file legends [file 41419_2024_6425_MOESM2_ESM.docx]

Supplementary file legends:

Supplementary file 1: 43 common interacting proteins were screened in the control and FBXO31 group through Venn diagram analysis.
