# Supplementary material for: FBXO31 is upregulated by METTL3 to promote pancreatic cancer progression via regulating SIRT2 ubiquitination and degradation
Source: Cell Death Dis. 2024 Jan 12;15(1):37. doi: 10.1038/s41419-024-06425-y (PMC10786907; doi:10.1038/s41419-024-06425-y)
Supplement: Supplementary file 3 — Supplementary figures [file 41419_2024_6425_MOESM3_ESM.pdf]

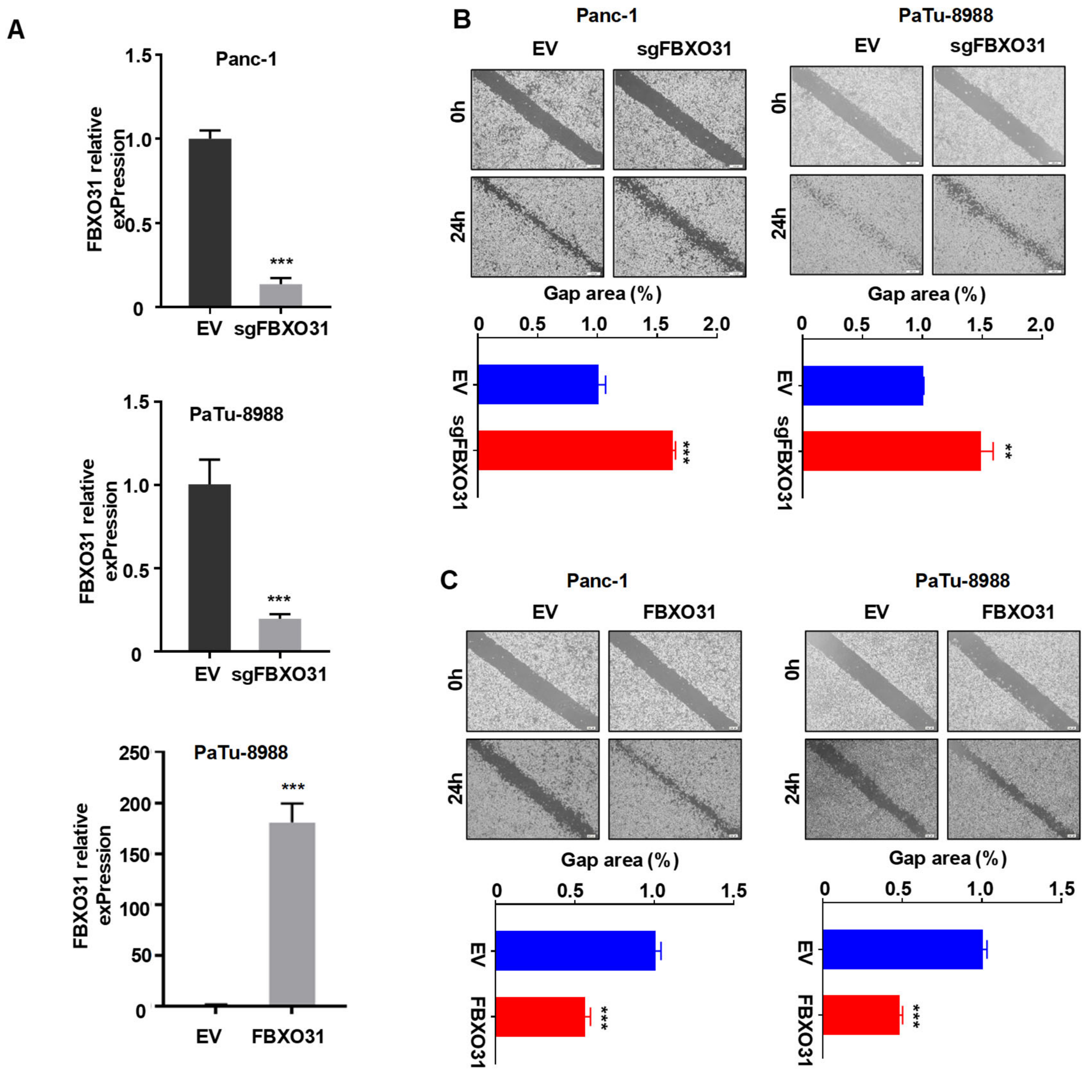

Figure S1. FBXO31 promotes cell migration.

A: qRT-PCR analysis to detect FBXO31 mRNA levels after FBXO31 depletion in Panc-1 and PaTu-8988 cells. qRT-PCR analysis to detect FBXO31 mRNA levels after FBXO31 overexpression in PaTu-8988 cells. ***p < 0.001 compared to control.

B: Wound healing assays to analyze cell migration capacity of Panc-1 and PaTu-8988 cells transfected with FBXO31 sgRNAs. **p<0.01, ***p<0.001 compared to control.

C: Wound healing assays to analyze cell migration capacity of Panc-1 and PaTu-8988 cells transfected with FBXO31 constructs. ***p<0.001 compared to control.

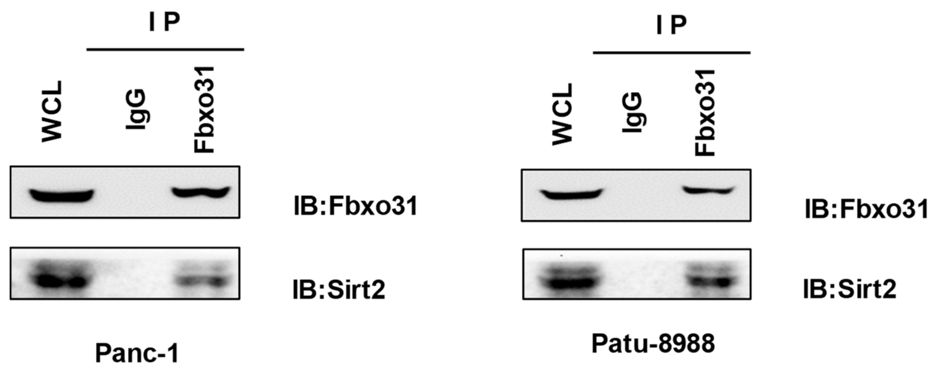

Figure S2. FBXO31 inhibited Sirt2 expression via interacting with Sirt2.
IB analysis of immunoprecipitates (IPs) and WCLs derived from Panc-1 cells and Patu-8988 cells. Cells were treated with 10 μM MG132 for 6 hours before harvesting.

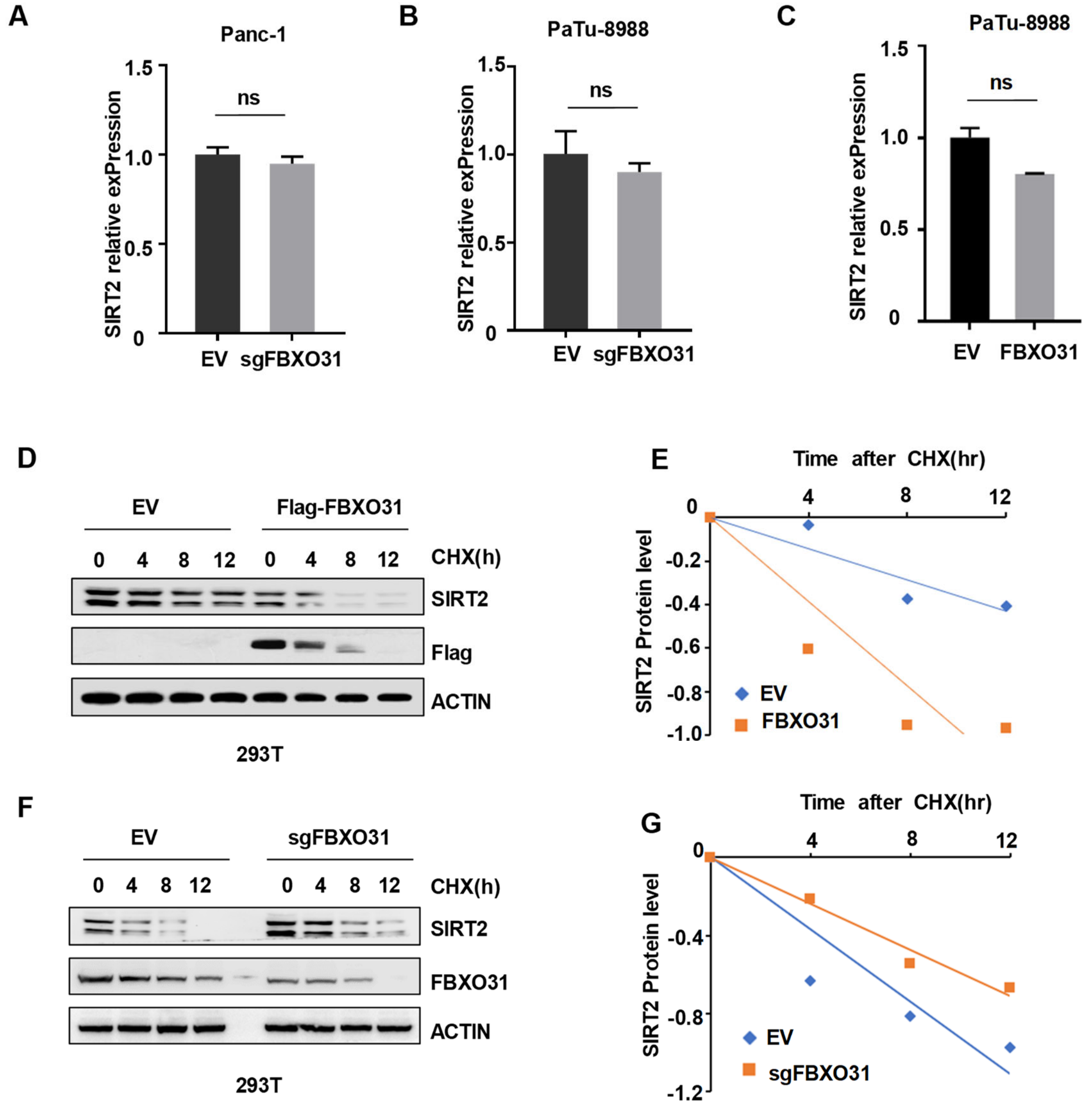

Figure S3. FBXO31 promotes the half-life of SIRT2.

A-B: qRT-PCR analysis to detect SIRT2 mRNA levels after FBXO31 depletion in Panc-1 (A) and PaTu-8988 (B) cells. ***p < 0.001 compared to control.

C: qRT-PCR analysis to detect FBXO31 and SIRT2 mRNA levels after FBXO31-overexpressing in PaTu-8988(B) cells. ***p < 0.001 compared to control.

D: IB analysis of WCLs derived from 293T cells transfected with FBXO31 constructs. Where indicated, 100 µg/ml cycloheximide (CHX) was added and cells were harvested at indicated time points.

E: SIRT2 protein abundance in (D) was quantified and plotted.

F: IB analysis of WCLs derived from 293T cells transfected with FBXO31 sgRNA. Where indicated, 100 µg/ml cycloheximide (CHX) was added and cells were harvested and lysed at indicated time points.

G: SIRT2 protein abundance in (F) was quantified and plotted.

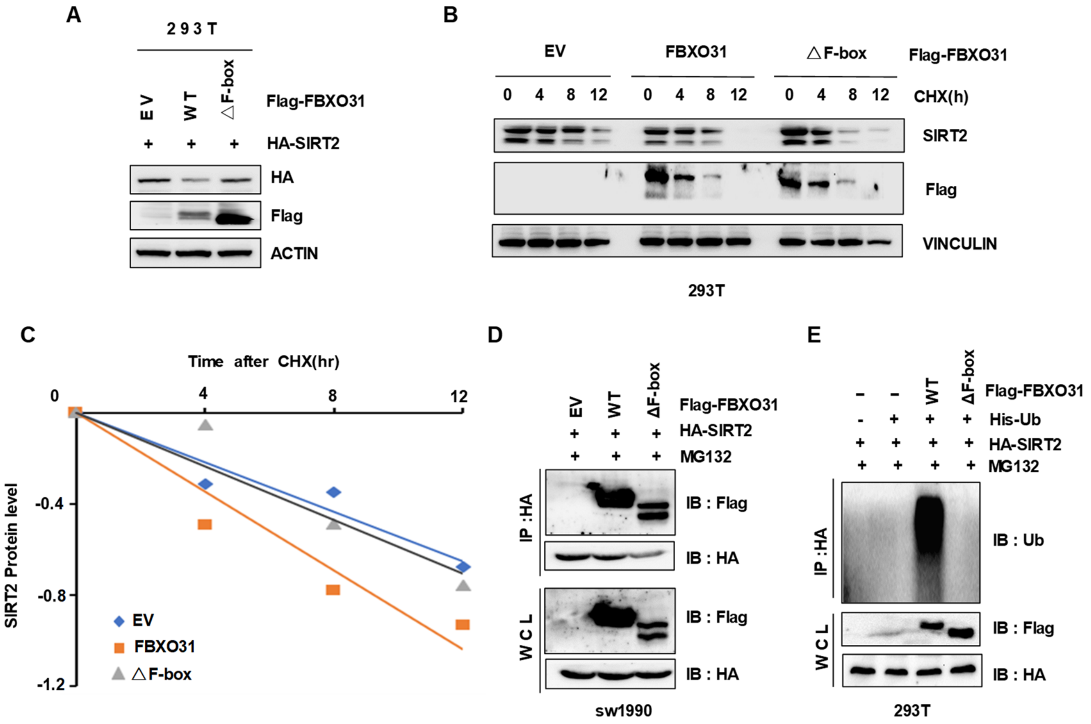

Figure S4. FBXO31-mediated destruction of SIRT2 is dependent on the F-box motif of FBXO31.

A: IB analysis of SIRT2 expression after transfection of FBXO31-WT and FBXO31-F-box plasmids in 293T cells.

B: IB analysis of WCLs derived from 293T cells transfected with FBXO31-WT or FBXO31-ΔF-box constructs. Where indicated, 100µg/ml cycloheximide (CHX) was added and cells were harvested and lysed at indicated time points.

C: SIRT2 protein abundance in (B) was quantified and plotted.

D: IB analysis of IPs and WCLs derived from sw1990 cells transfected with the FBXO31-WT and FBXO31-ΔF-box plasmids.

Cells were treated with 10 µM MG132 for 6 h before harvesting.

E: IB analysis of ubiquitination products and WCLs derived from 293T cells transfected with the FBXO31-WT and FBXO31-ΔF-box plasmids. Cells were treated with 10 µM MG132 for 6 h before harvesting.

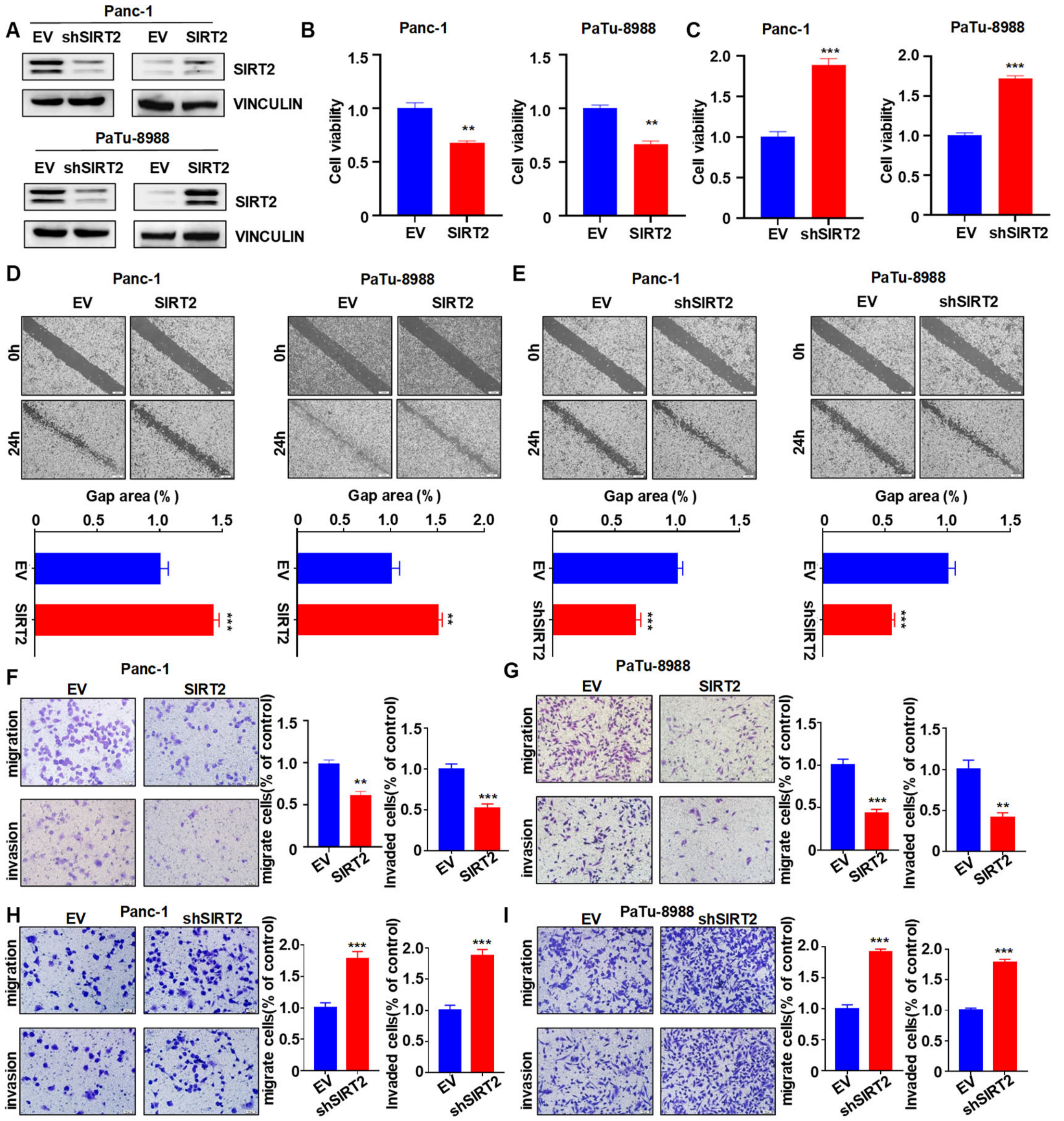

Figure S5: SIRT2 inhibits cell viability, migration and invasion in PC cells.

A: IB analysis of WCLs derived from the Panc-1 and PaTu-8988 cells transfected with the indicated plasmids.

B-C : MTT assays to detect cell proliferation in Panc-1 and PaTu-8988 cells transfected with SIRT2 shRNAs or SIRT2 constructs. **$p < 0.01$ compared to control, ***$p < 0.001$ compared to control.

D-E: Wound healing assays to analyze cell migration capacity of Panc-1 and PaTu-8988 cells transfected with SIRT2 shRNAs or SIRT2 constructs. **$p < 0.01$ compared to control, ***$p < 0.001$ compared to control.

F-I: Transwell assays to analyze cell migration and invasion capacity of Panc-1 and PaTu-8988 cells transfected with SIRT2 shRNAs or SIRT2 constructs. **$p < 0.01$ compared to control, ***$p < 0.001$ compared to control.

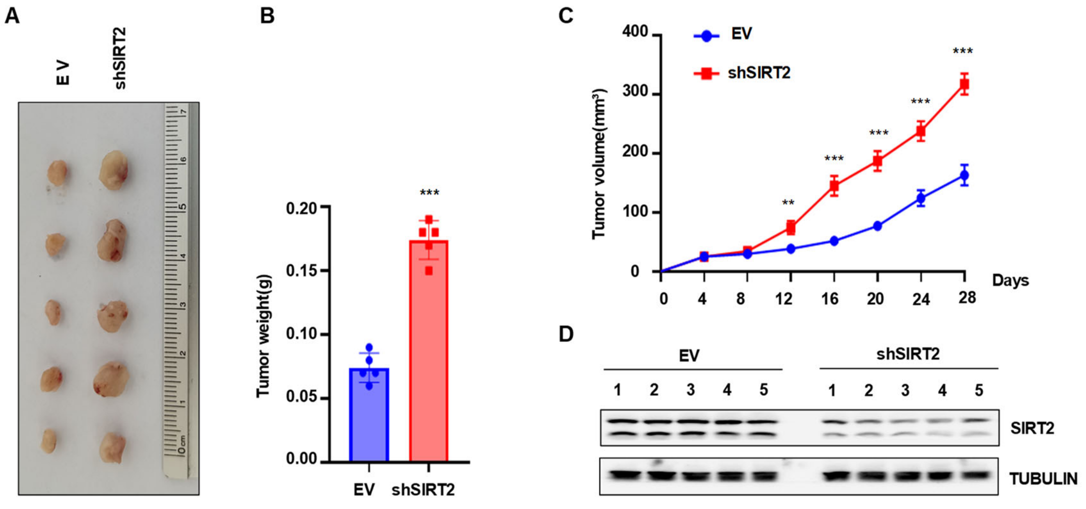

Figure S6: Depletion of SIRT2 promotes tumor growth in mice.

A: Pictures of tumor masses dissected from SIRT2-knockdown xenograft mouse models. Panc-1 cells with stable SIRT2-knockdown and the control cells were injected subcutaneously into the BALB/c-nu/nu mice to establish xenograft mouse models.

B-C: Tumor weights and tumor volumes of dissected tumor mass in (A).

D: IB analysis of the SIRT2 protein levels in the dissected tumors.

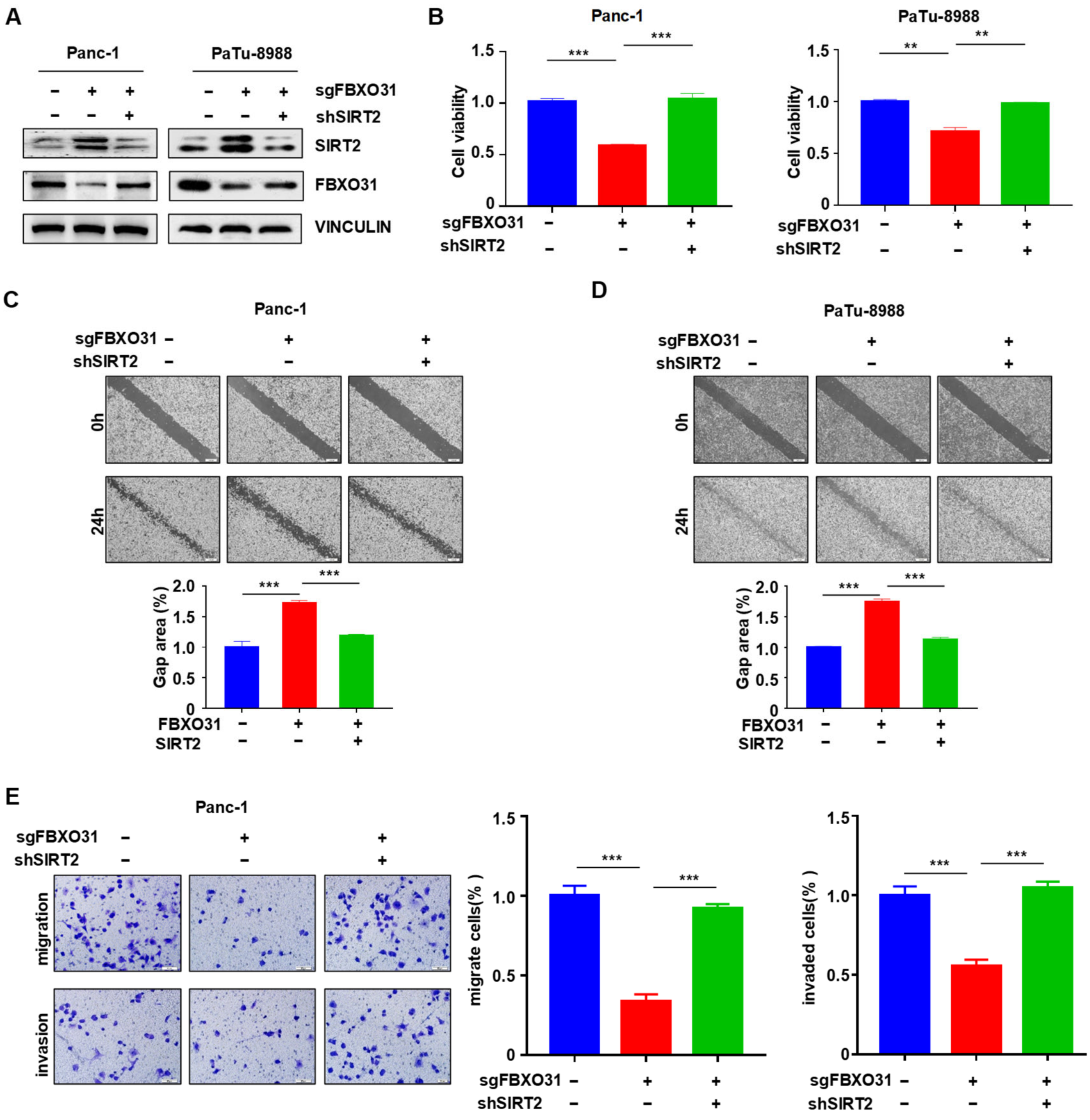

Figure S7: SIRT2 is required for FBXO31-mediated promotion effect of cell viability, migration and invasion in PC.

A: IB analysis of WCLs derived from Panc-1 and PaTu-8988 cells transfected with the indicated plasmids.

B: MTT assays to detect cell proliferation in Panc-1 and PaTu-8988 cells transfected with the indicated plasmids. **p < 0.01 compared to control, ***p < 0.001 compared to control.

C-D: Wound healing assays to analyze cell migration capacity of Panc-1 and PaTu-8988 cells transfected with the indicated plasmids. ***p < 0.001 compared to control.

E: Transwell assays to analyze cell migration and invasion capacity of Panc-1 and PaTu-8988 cells transfected with the indicated plasmids. ***p < 0.001 compared to control.

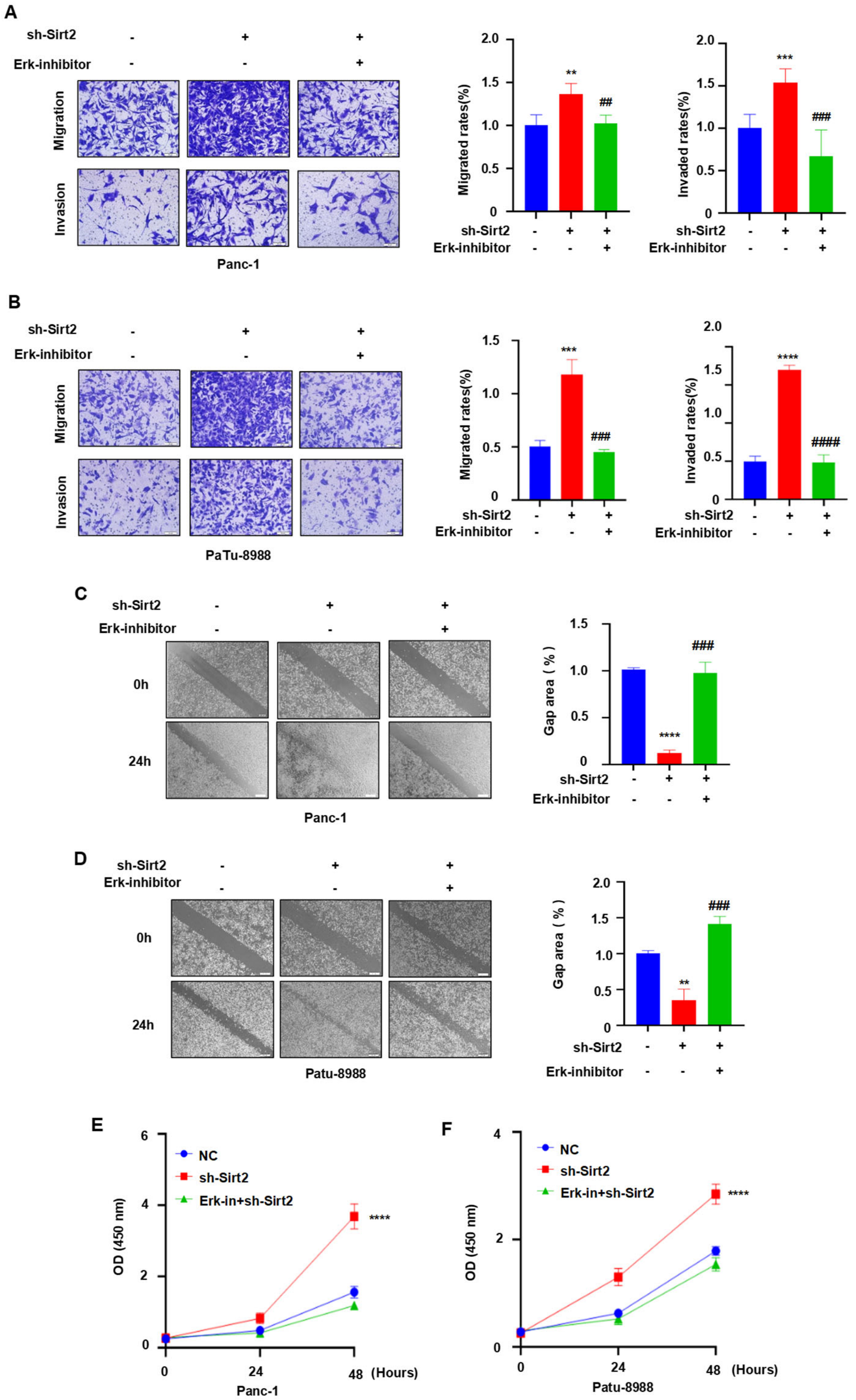

Figure S8: SIRT2 performed its functions in part via the ERK pathway.

A-B: Transwell assays to analyze cell migration and invasion capacity of Panc-1 and PaTu-8988 cells treated with sh-SIRT2 and Erk inhibitor.

C-D: Wound healing assay to analyze cell migration of Panc-1 and PaTu-8988 cells treated with sh-SIRT2 and Erk inhibitor.

E-F: CCK-8 assay to analyze cell viability of Panc-1 and PaTu-8988 cells treated with sh-SIRT2 and Erk inhibitor. **$p < 0.01$, ***$p < 0.001$, ****$p < 0.0001$ compared to control. $^{\#\#}p < 0.01$, $^{\#\#\#}p < 0.001$ compared to sh-Sirt2.

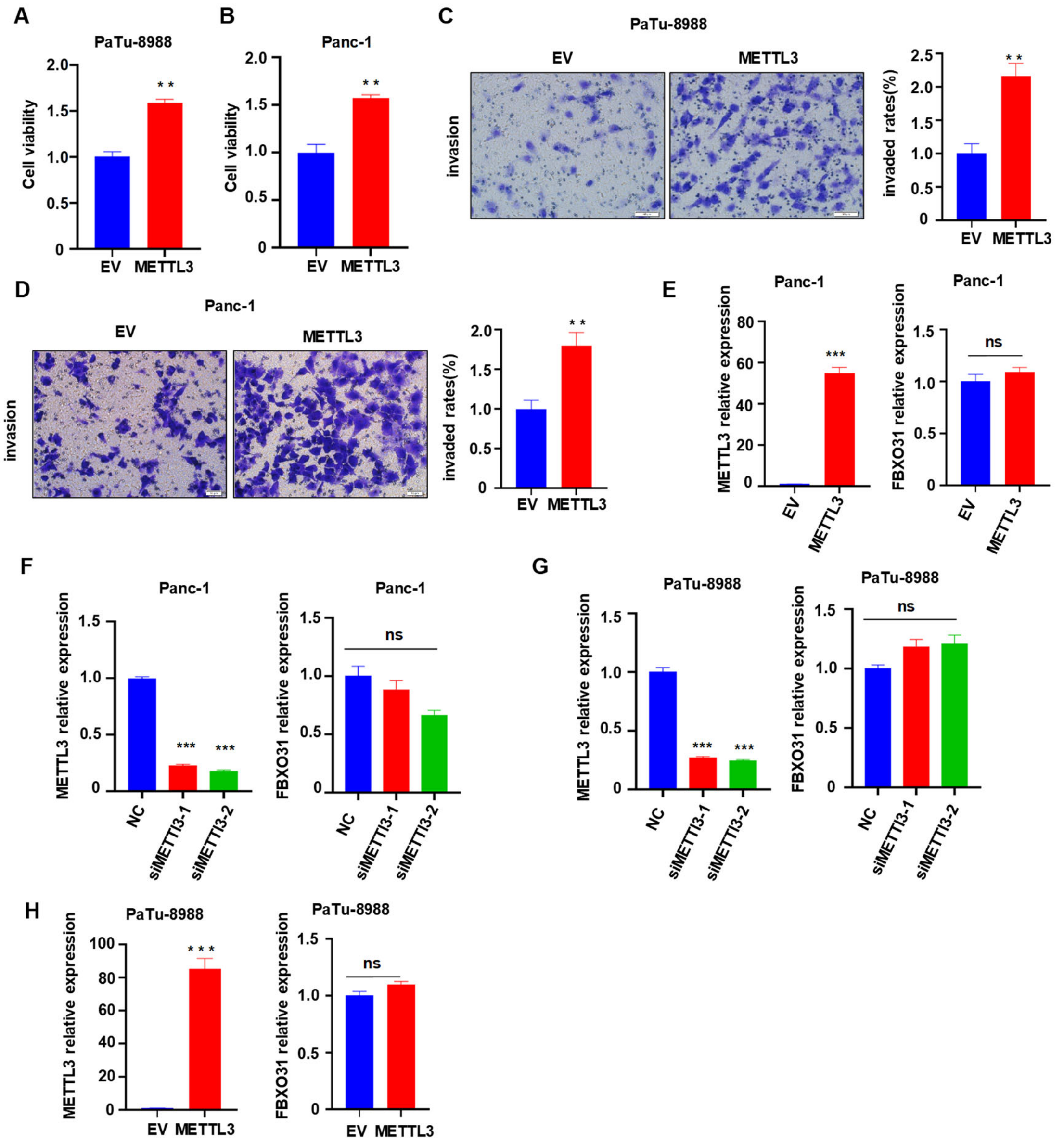

Figure S9: METTL3 promotes cell viability and invasion in PC.

A-B: MTT assays to detect cell proliferation in Panc-1 and PaTu-8988 cells transfected with METTL3 constructs. **$p < 0.01$ compared to control.

C-D: Transwell assays to analyze cell migration and invasion capacity of Panc-1 and PaTu-8988 cells transfected with METTL3 constructs. **$p < 0.01$ compared to control.

E-F: qRT-PCR analysis to detect METTL3 and FBXO31 mRNA levels after METTL3 overexpressing (E) or depletion (F) in Panc-1 cells. ***$p < 0.001$ compared to control.

G-H: qRT-PCR analysis to detect METTL3 and FBXO31 mRNA levels after METTL3 overexpressing (G) or depletion (H) in PaTu-8988 cells. ***$p < 0.001$ compared to control.

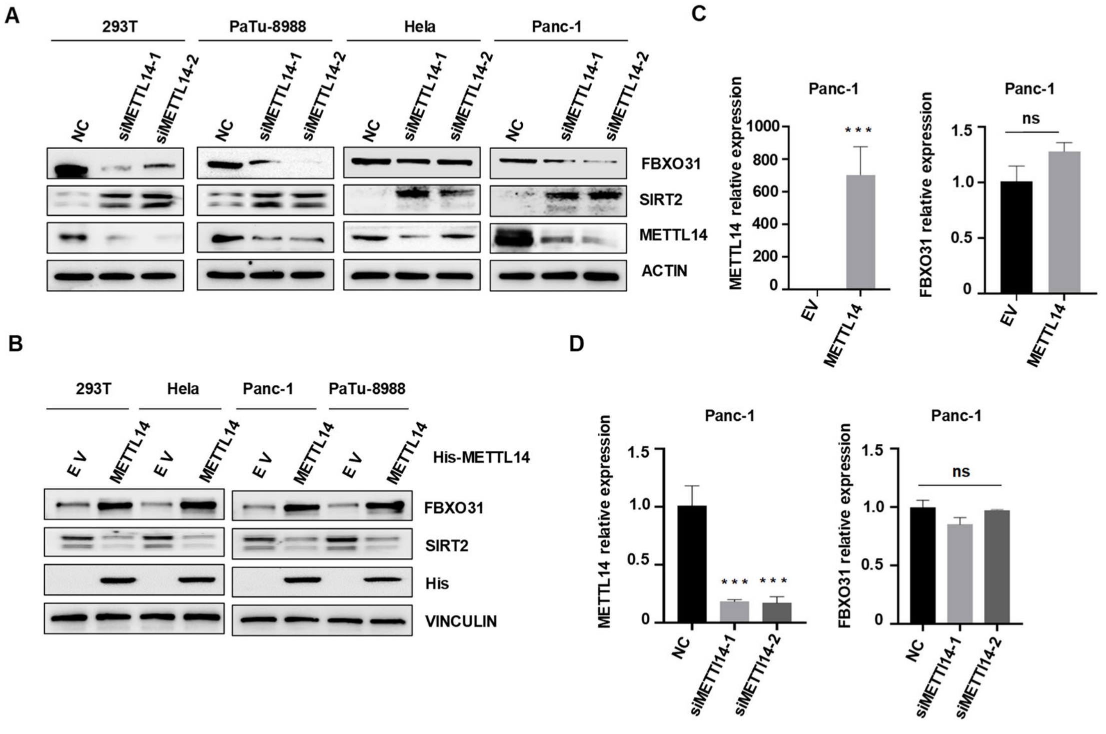

Figure S10: METTL14 affects the expression of FBXO31 and SIRT2 in PC cells.
A-B: IB analysis of WCLs derived from Panc-1, PaTu-8988, HeLa and 293T cells transfected with the indicated plasmids.
C-D: qRT-PCR analysis to detect METTL14 and FBXO31 mRNA levels after METTL14 overexpressing (C) or depletion (D) in Panc-1 cells. ***$p < 0.001$ compared to control.
