# Supplementary material for: FBXO31 is upregulated by METTL3 to promote pancreatic cancer progression via regulating SIRT2 ubiquitination and degradation
Source: Cell Death Dis. 2024 Jan 12;15(1):37. doi: 10.1038/s41419-024-06425-y (PMC10786907; doi:10.1038/s41419-024-06425-y)
Supplement: Supplementary file 4 — Original WB Images [file 41419_2024_6425_MOESM4_ESM.pdf]

Fig 1

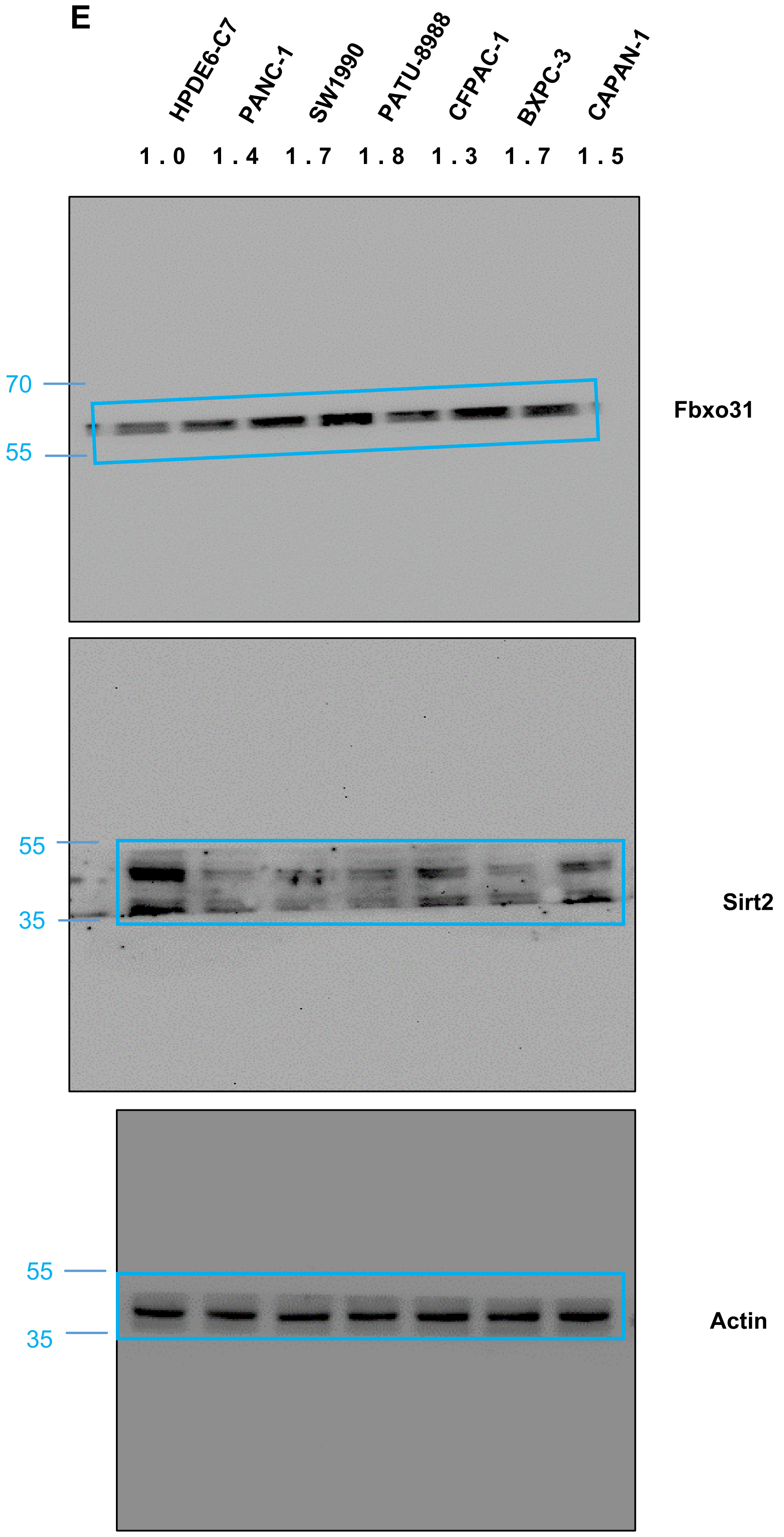

E
HPDE6-C7
PANC-1
SW1990
PATU-8988
CFPAC-1
BXPC-3
CAPAN-1
1.0 1.4 1.7 1.8 1.3 1.7 1.5
70
55
Fbxo31
55
35
Sirt2
55
35
Actin

Fig 2

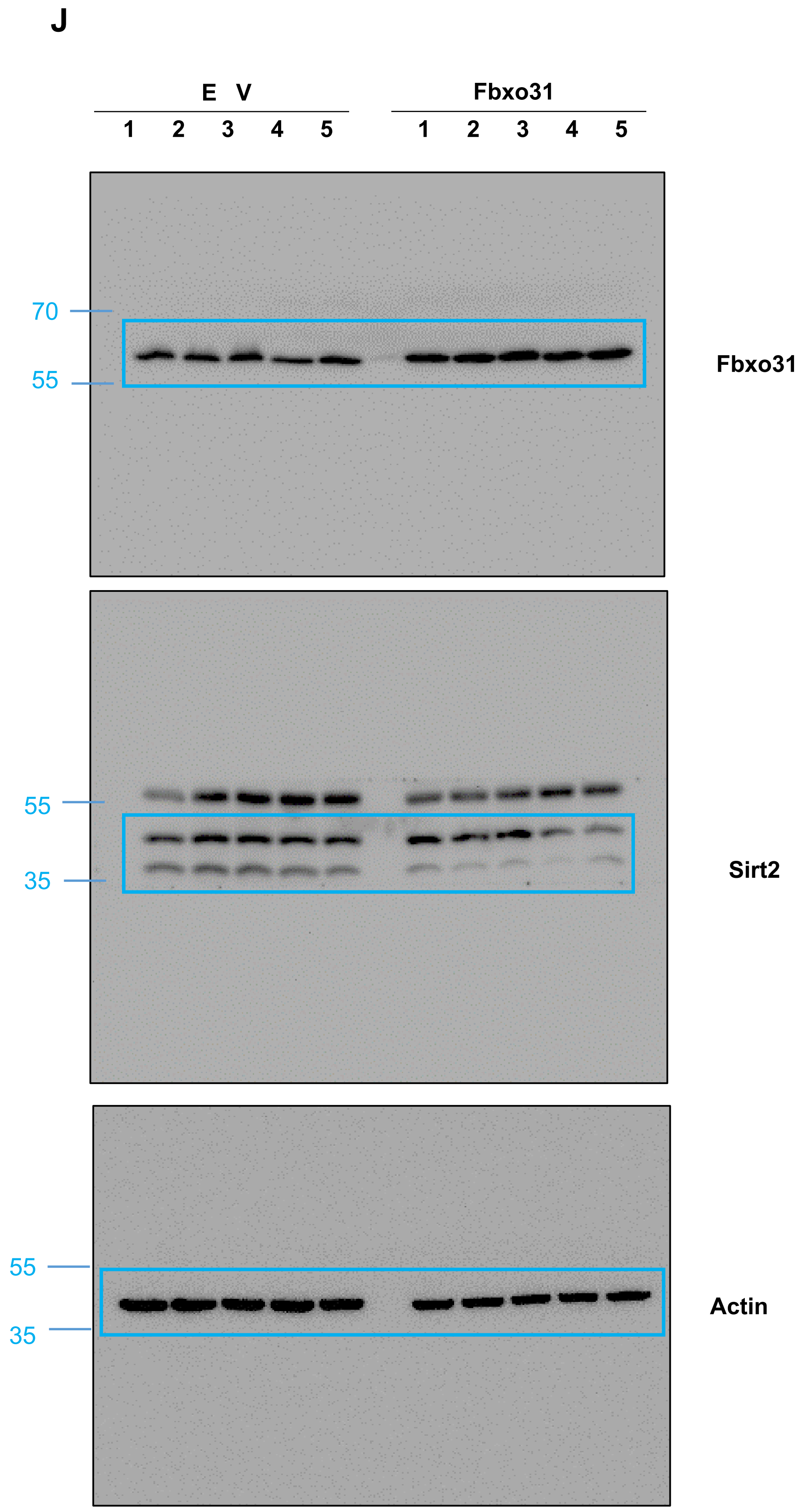

J
E V
Fbxo31
1 2 3 4 5
1 2 3 4 5
70
55
Fbxo31
55
35
Sirt2
55
35
Actin

Fig 3

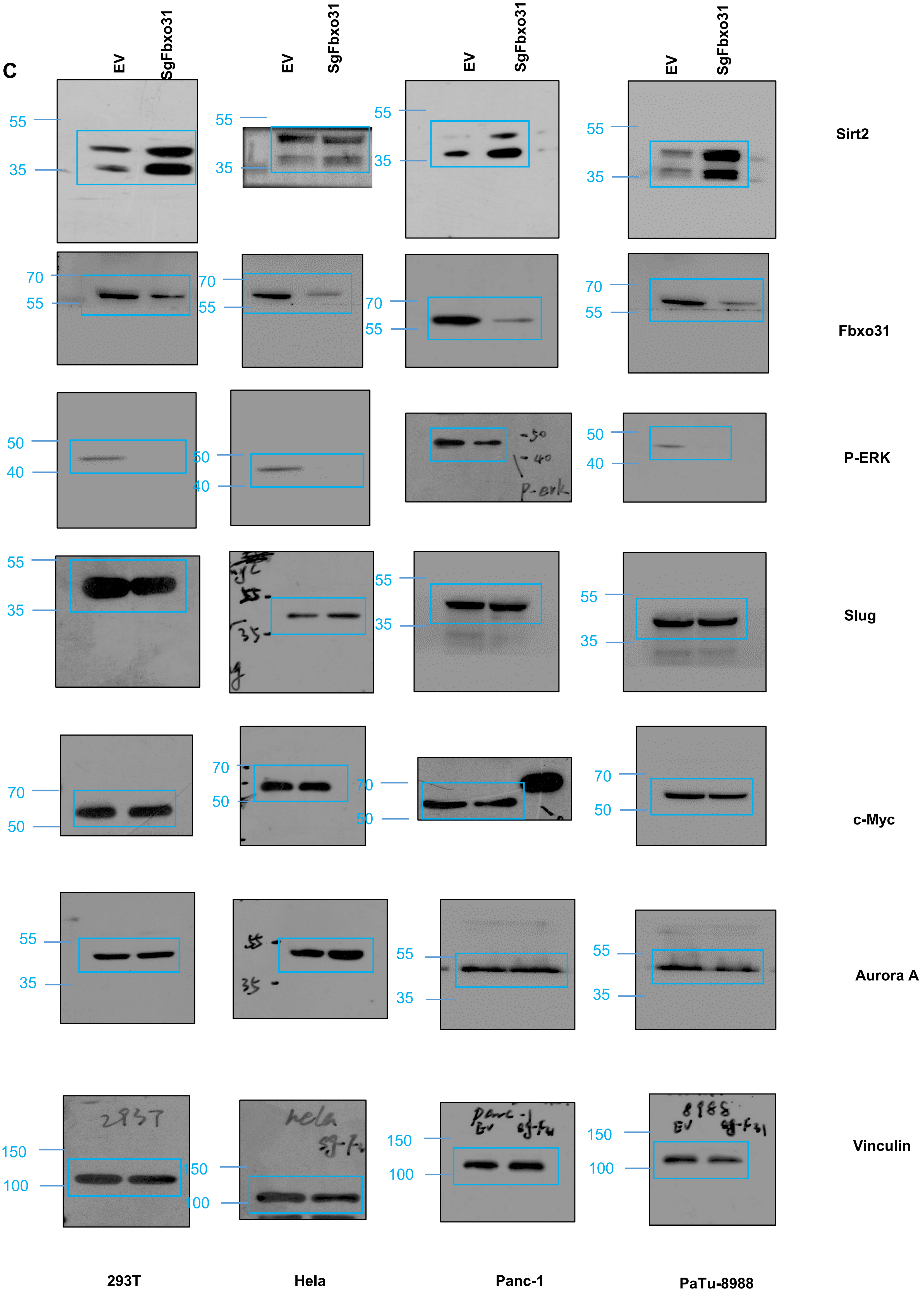
C
EV
SgFbxo31
55
35
70
50
40
150
100
Sirt2
Fbxo31
P-ERK
Slug
c-Myc
Aurora A
Vinculin
293T
Hela
Panc-1
PaTu-8988

Fig 3

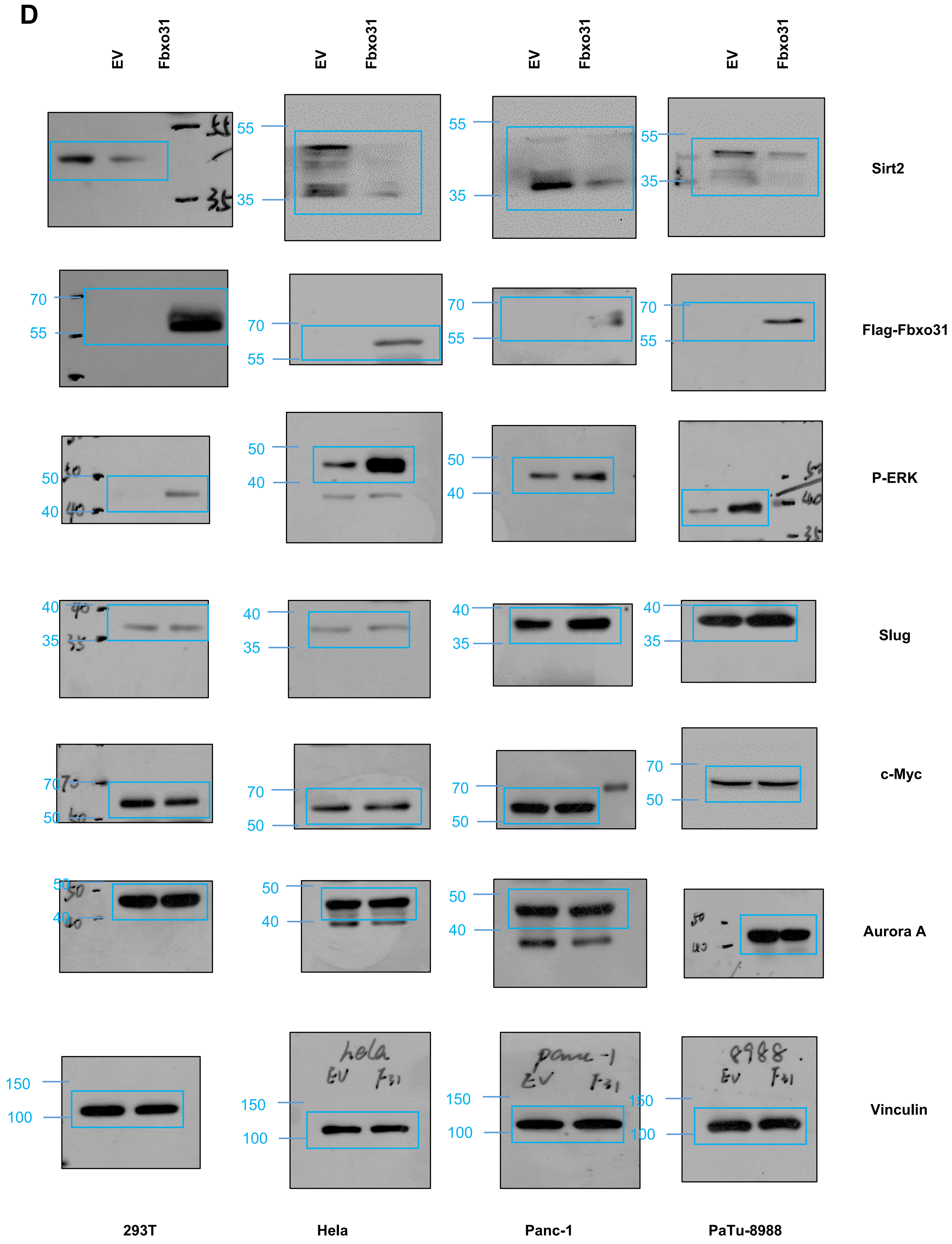
D
EV
Fbxo31
Sirt2
Flag-Fbxo31
P-ERK
Slug
c-Myc
Aurora A
Vinculin
293T
Hela
Panc-1
PaTu-8988
55
35
70
50
40
150
100

Fig 3

E

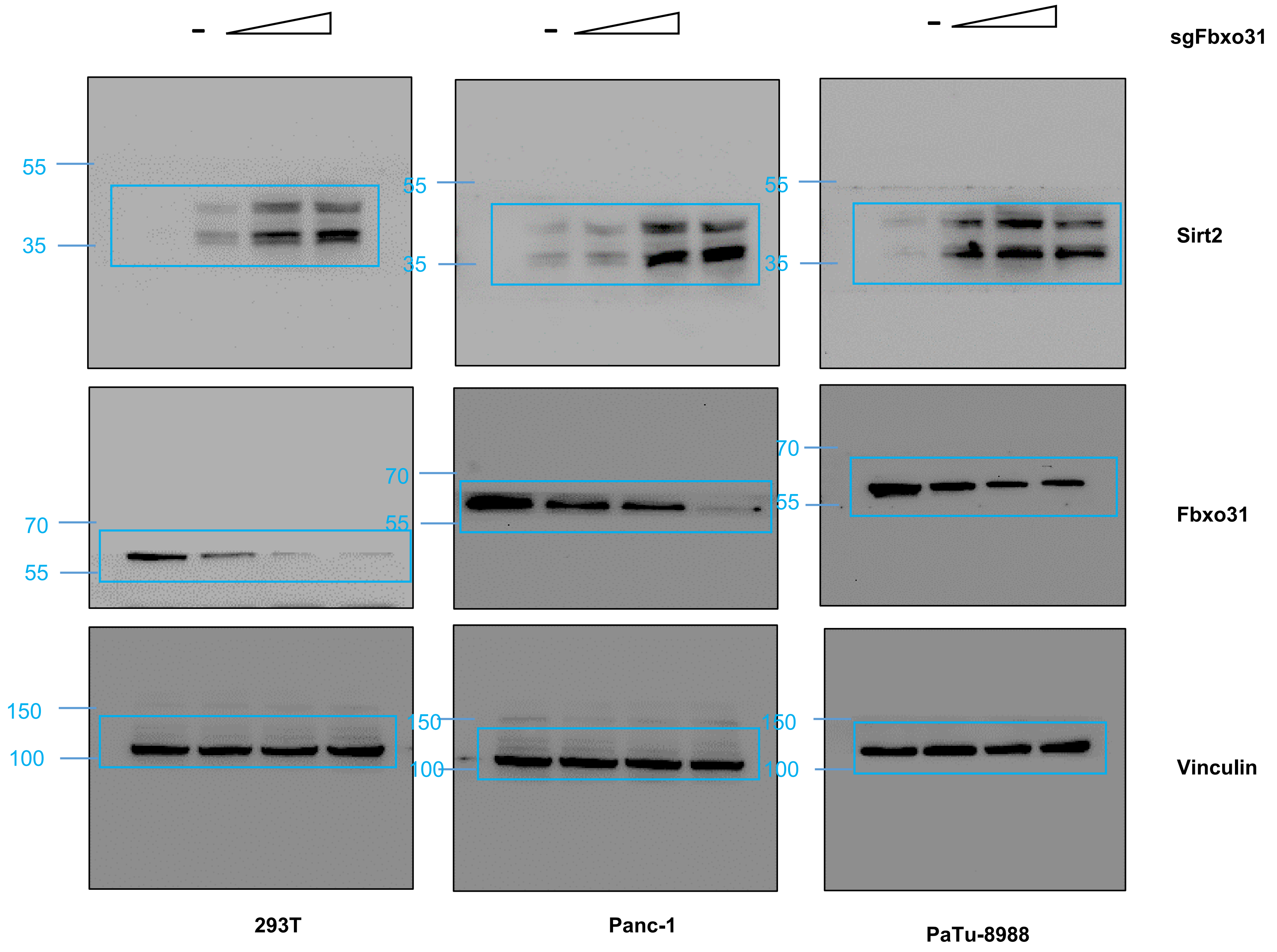
sgFbxo31
Sirt2
Fbxo31
Vinculin
55
35
70
150
100
293T
Panc-1
PaTu-8988

F

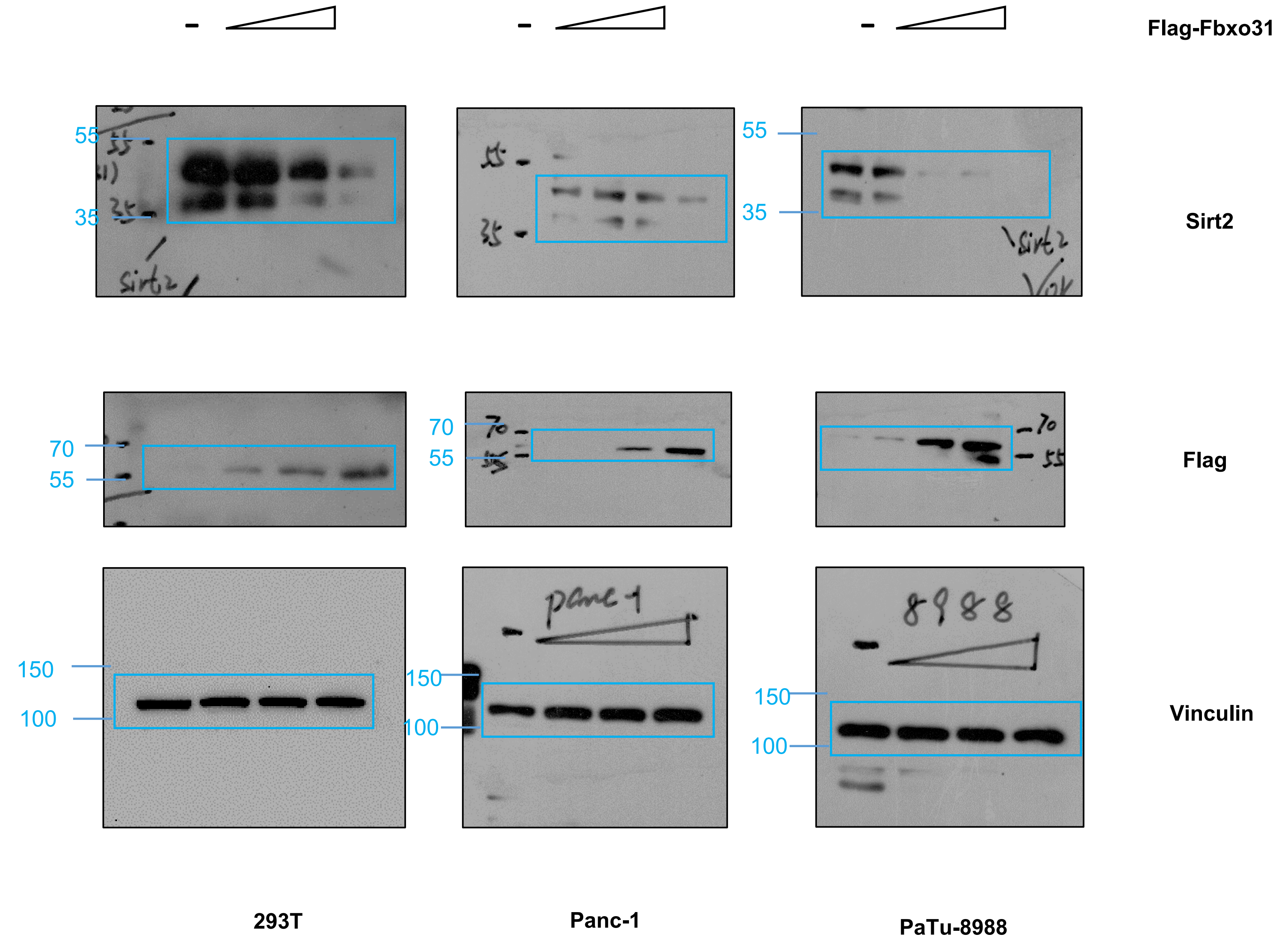
Flag-Fbxo31
Sirt2
Flag
Vinculin
55
35
70
150
100
293T
Panc-1
PaTu-8988

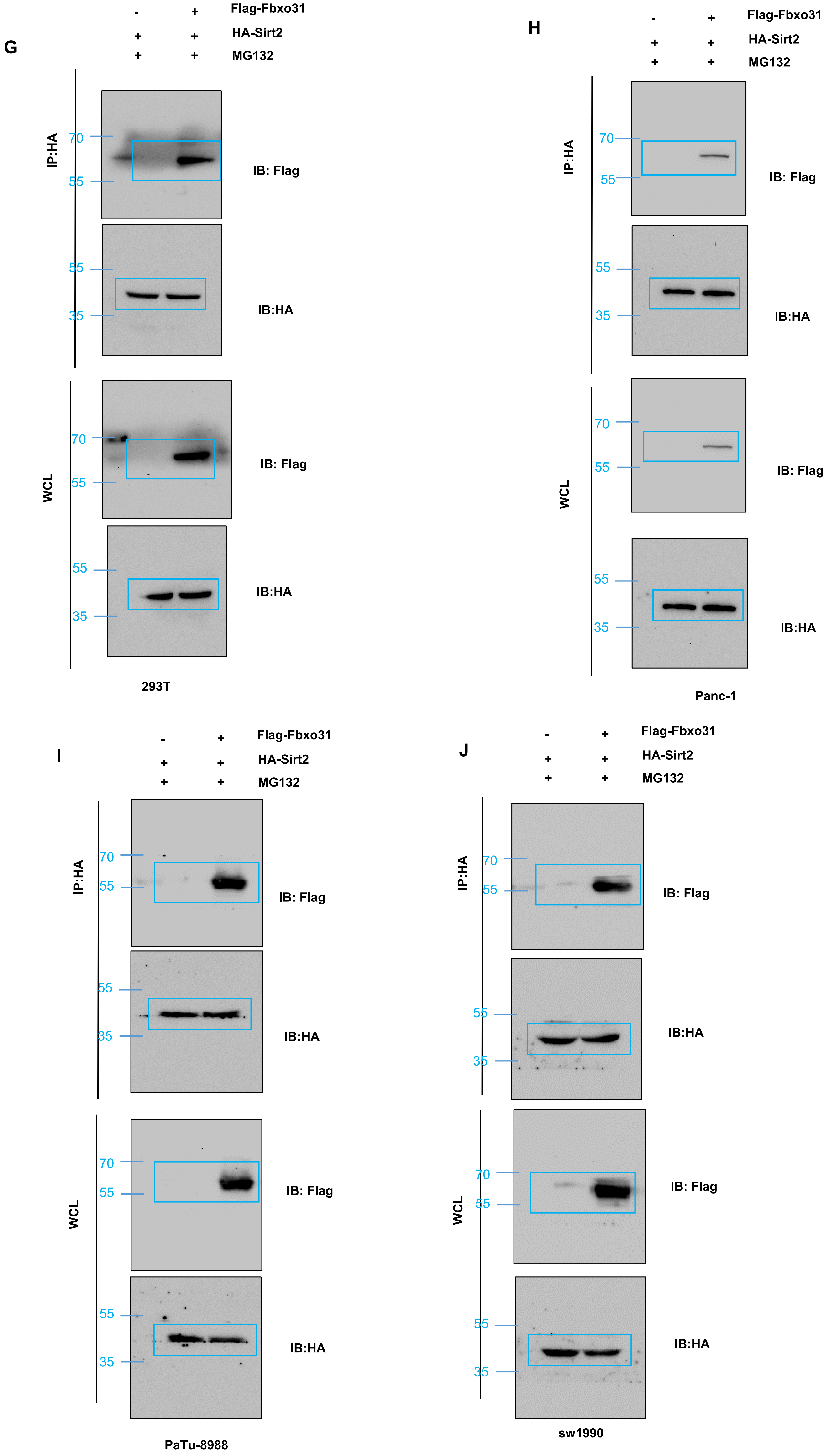
G
- + Flag-Fbxo31
+ + HA-Sirt2
+ + MG132
IP:HA
70
55
IB: Flag
55
35
IB:HA
WCL
70
55
IB: Flag
55
35
IB:HA
293T
H
- + Flag-Fbxo31
+ + HA-Sirt2
+ + MG132
IP:HA
70
55
IB: Flag
55
35
IB:HA
WCL
70
55
IB: Flag
55
35
IB:HA
Panc-1
I
- + Flag-Fbxo31
+ + HA-Sirt2
+ + MG132
IP:HA
70
55
IB: Flag
55
35
IB:HA
WCL
70
55
IB: Flag
55
35
IB:HA
PaTu-8988
J
- + Flag-Fbxo31
+ + HA-Sirt2
+ + MG132
IP:HA
70
55
IB: Flag
55
35
IB:HA
WCL
70
55
IB: Flag
55
35
IB:HA
sw1990

Fig 4

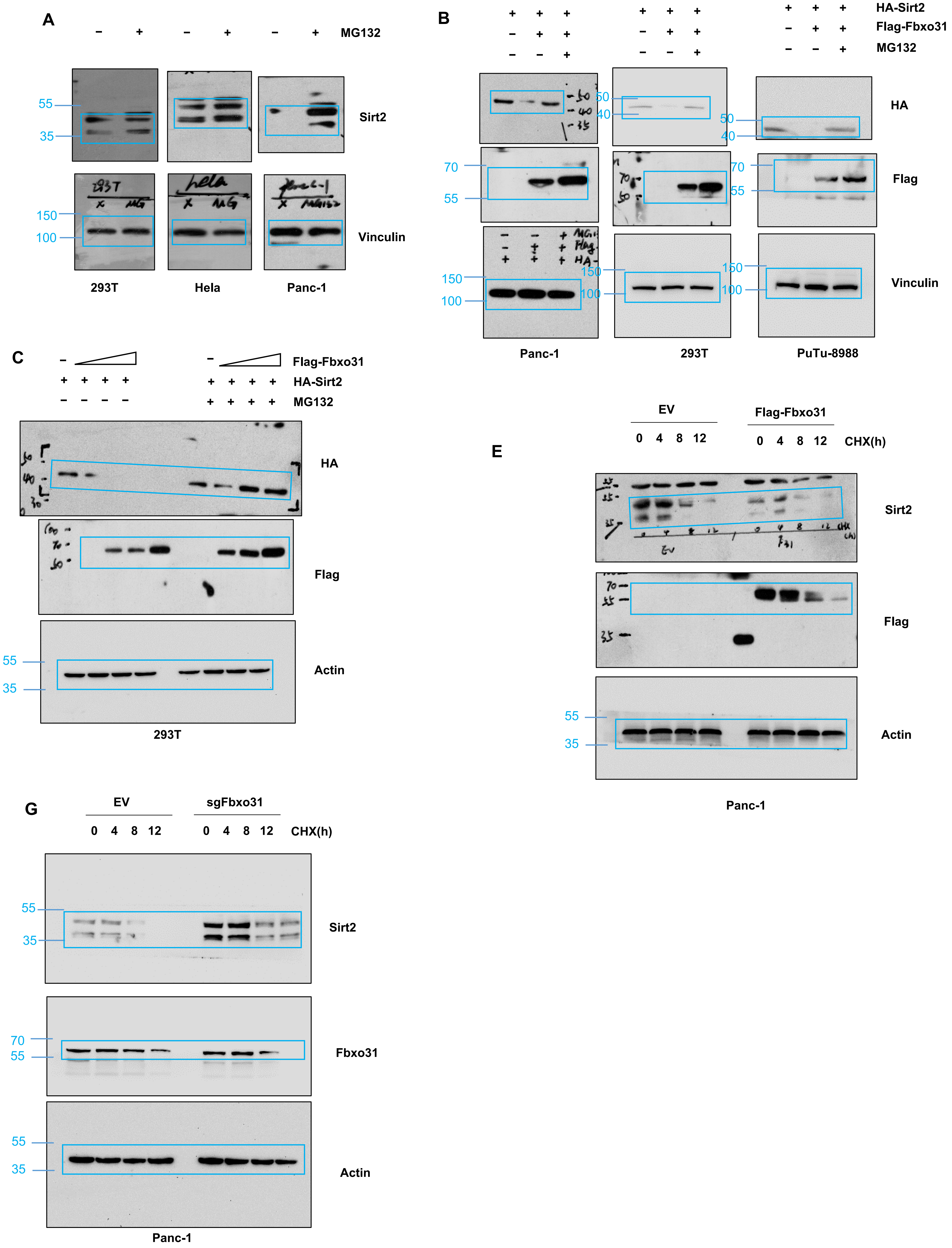

A
MG132
55
35
Sirt2
150
100
Vinculin
293T
Hela
Panc-1
B
HA-Sirt2
Flag-Fbxo31
MG132
50
40
HA
70
55
Flag
150
100
Vinculin
Panc-1
293T
PuTu-8988
C
Flag-Fbxo31
HA-Sirt2
MG132
HA
Flag
55
35
Actin
293T
EV
Flag-Fbxo31
0 4 8 12
0 4 8 12
CHX(h)
E
Sirt2
Flag
55
35
Actin
Panc-1
G
EV
sgFbxo31
0 4 8 12
0 4 8 12
CHX(h)
55
35
Sirt2
70
55
Fbxo31
55
35
Actin
Panc-1

Fig 4

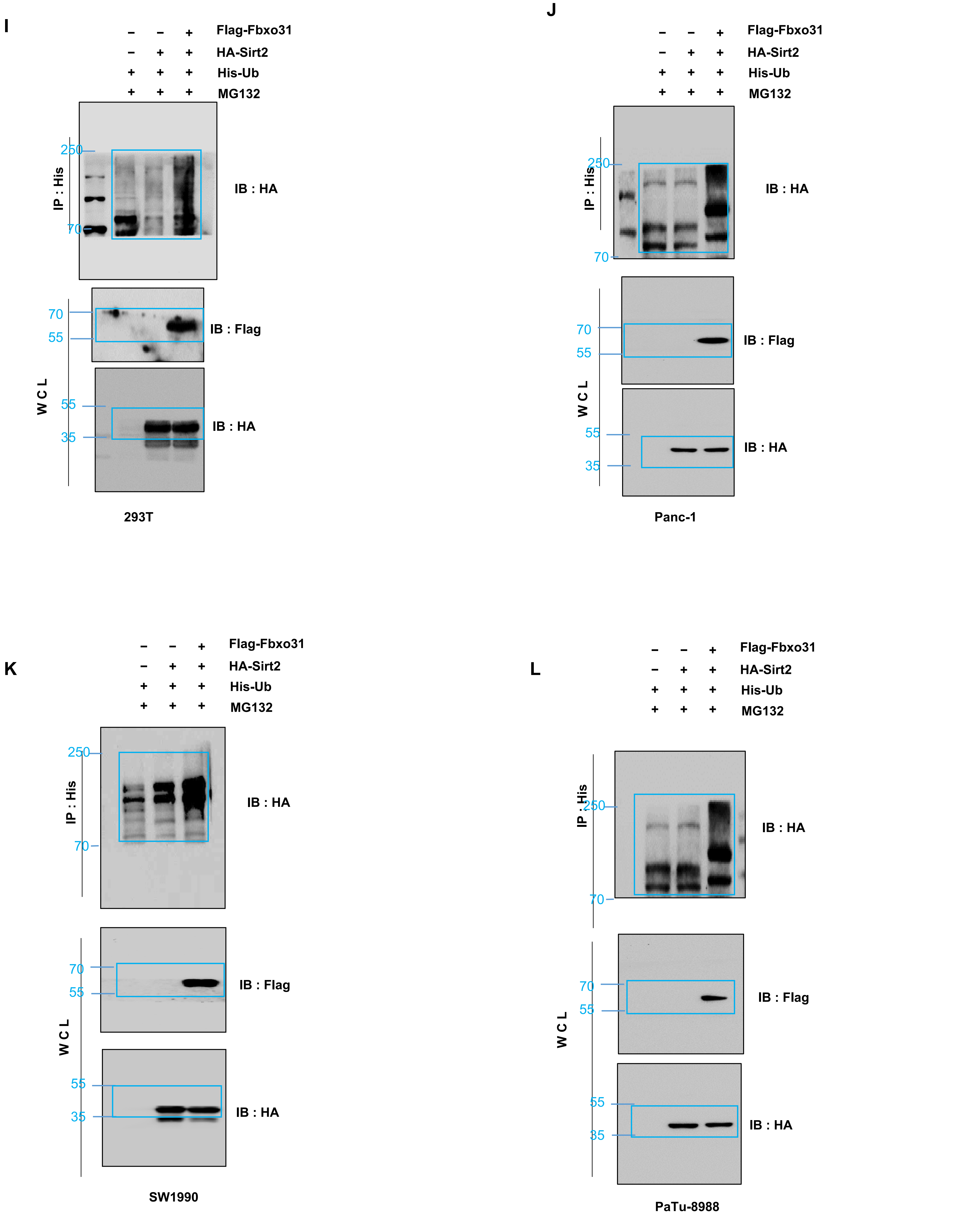
I
J
K
L
Flag-Fbxo31
HA-Sirt2
His-Ub
MG132
IP : His
IB : HA
IB : Flag
W C L
250
70
55
35
293T
Panc-1
SW1990
PaTu-8988

Fig 5

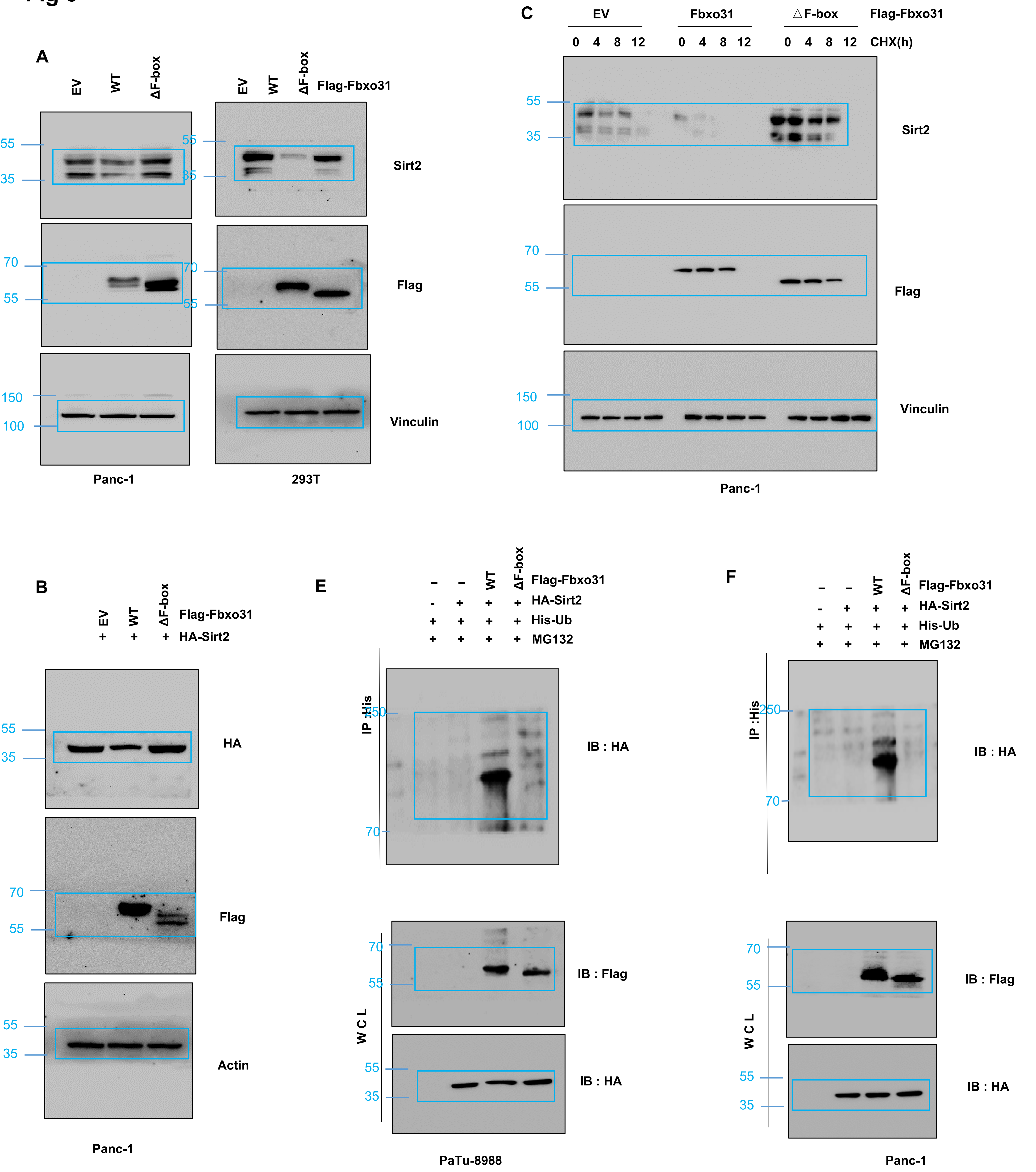
A
EV
WT
ΔF-box
Flag-Fbxo31
Sirt2
Flag
Vinculin
Panc-1
293T
C
EV
Fbxo31
△F-box
Flag-Fbxo31
0 4 8 12
CHX(h)
Sirt2
Flag
Vinculin
Panc-1
B
HA-Sirt2
HA
Actin
Panc-1
E
HA-Sirt2
His-Ub
MG132
IP :His
IB : HA
W C L
IB : Flag
IB : HA
PaTu-8988
F
Panc-1

Fig 5

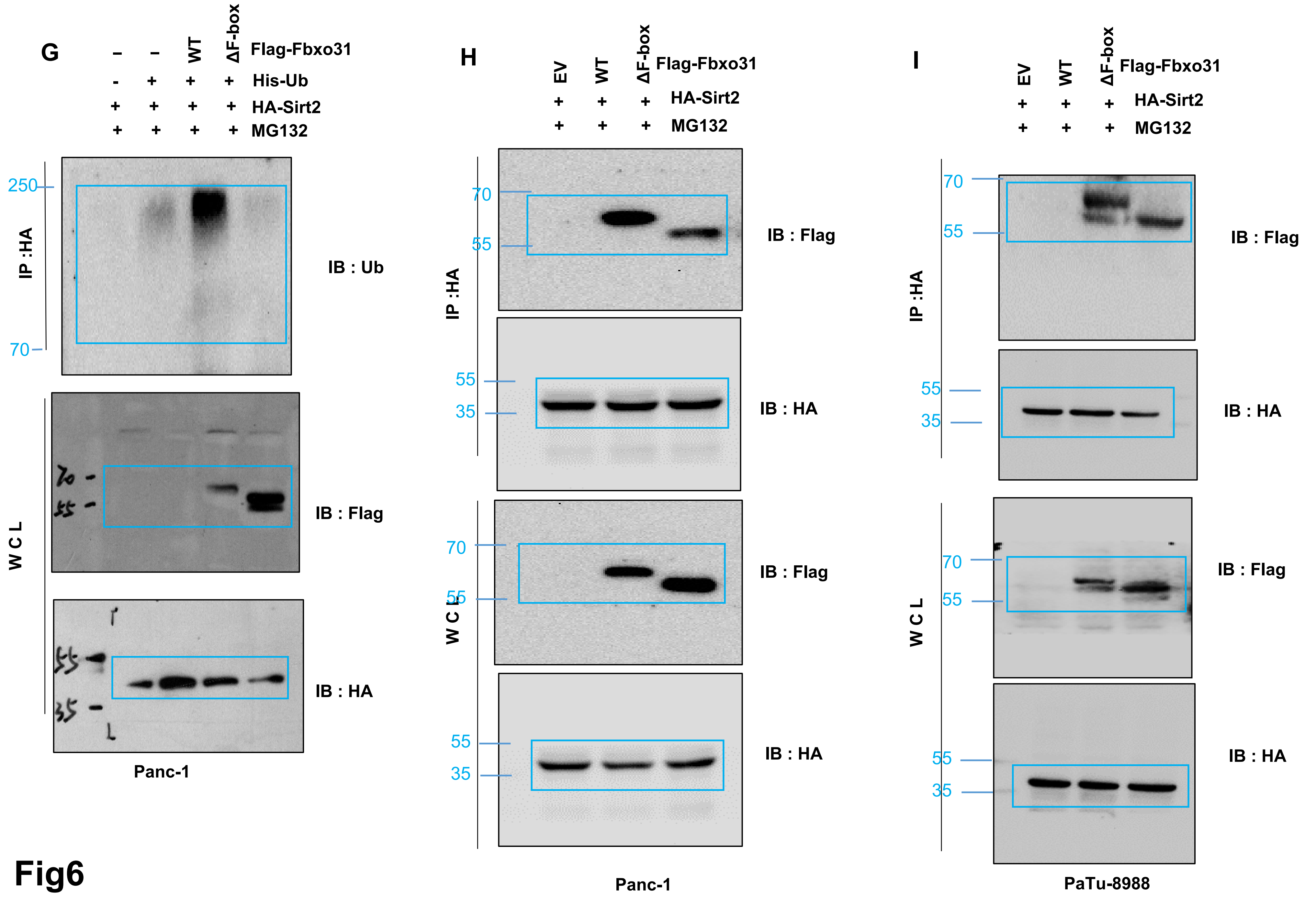
G
– – WT ΔF-box Flag-Fbxo31
- + + + His-Ub
+ + + + HA-Sirt2
+ + + + MG132
250
70
IP :HA
IB : Ub
W C L
70
55
IB : Flag
55
35
IB : HA
Panc-1
H
EV WT ΔF-box Flag-Fbxo31
+ + + HA-Sirt2
+ + + MG132
70
55
IB : Flag
IP :HA
55
35
IB : HA
70
55
IB : Flag
W C L
55
35
IB : HA
Panc-1
I
EV WT ΔF-box Flag-Fbxo31
+ + + HA-Sirt2
+ + + MG132
70
55
IB : Flag
IP :HA
55
35
IB : HA
70
55
IB : Flag
W C L
55
35
IB : HA
PaTu-8988

Fig6

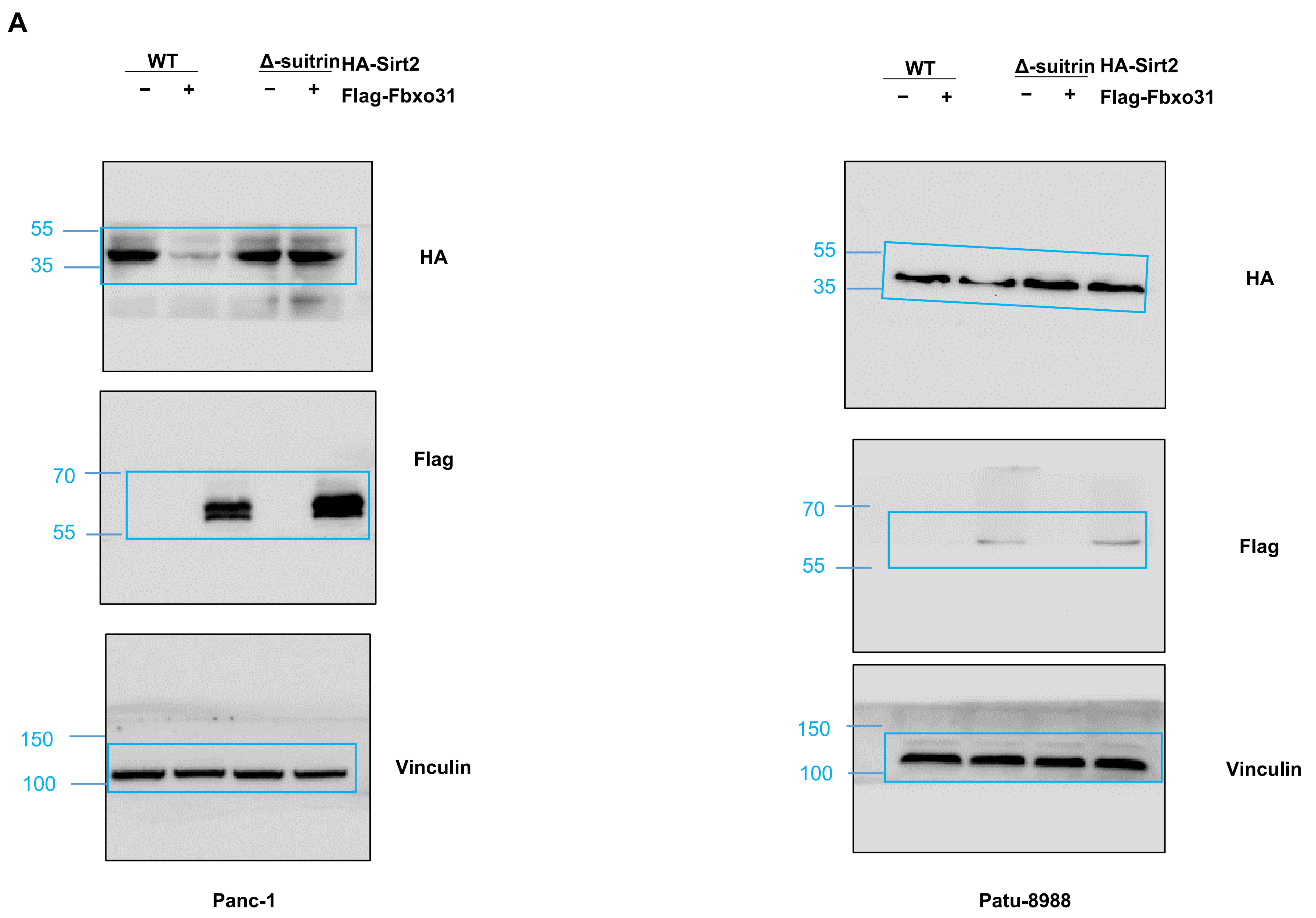
A
WT Δ-suitrin HA-Sirt2
– + – + Flag-Fbxo31
55
35
HA
70
55
Flag
150
100
Vinculin
Panc-1
WT Δ-suitrin HA-Sirt2
– + – + Flag-Fbxo31
55
35
HA
70
55
Flag
150
100
Vinculin
Patu-8988

Fig 6

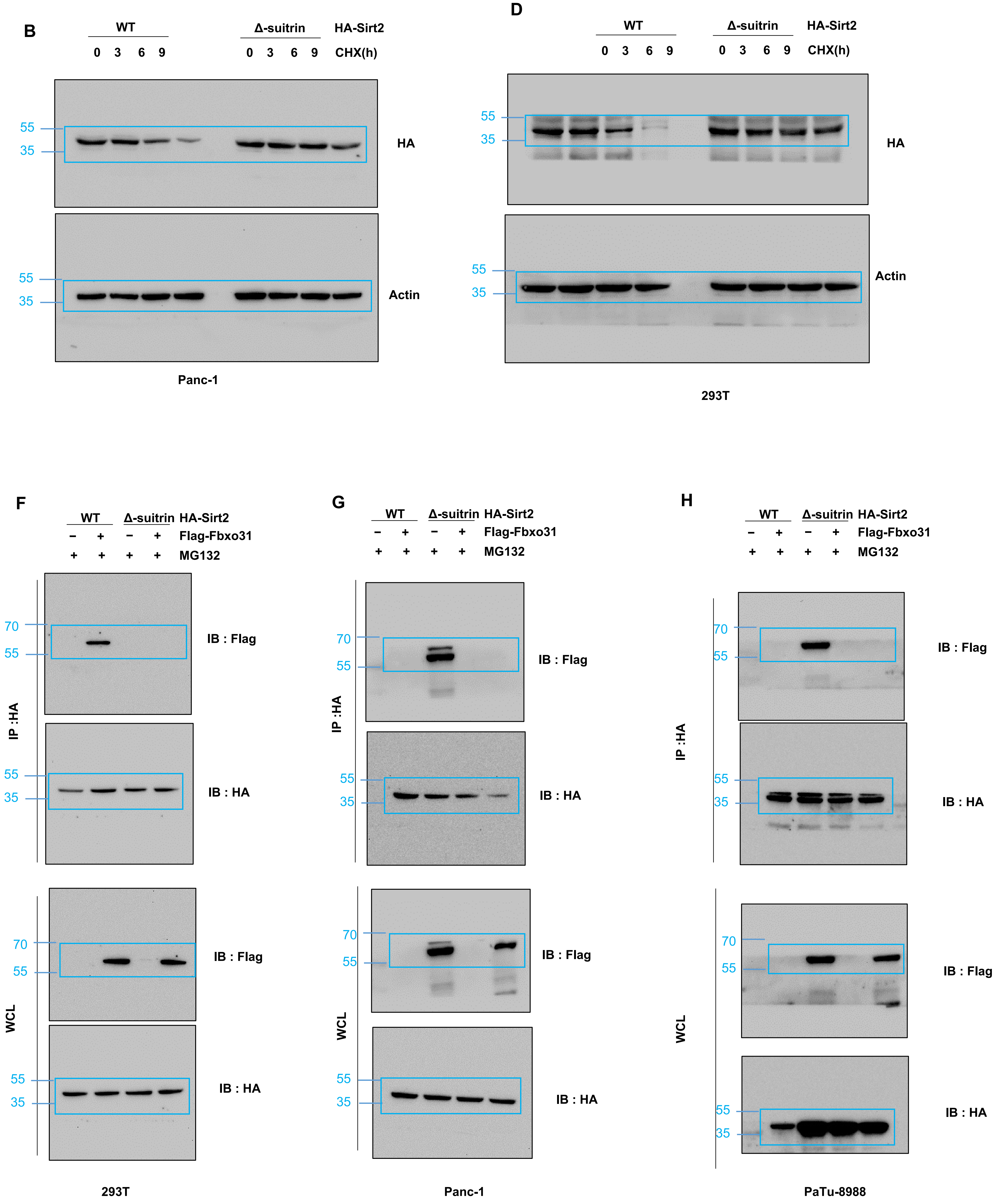

B
WT
Δ-suitrin
HA-Sirt2
0 3 6 9
0 3 6 9
CHX(h)
55
35
HA
Actin
Panc-1
D
WT
Δ-suitrin
HA-Sirt2
0 3 6 9
0 3 6 9
CHX(h)
HA
Actin
293T
F
G
H
WT
Δ-suitrin HA-Sirt2
− + − + Flag-Fbxo31
+ + + + MG132
IP :HA
WCL
70
IB : Flag
IB : HA
293T
Panc-1
PaTu-8988

Fig 6

I

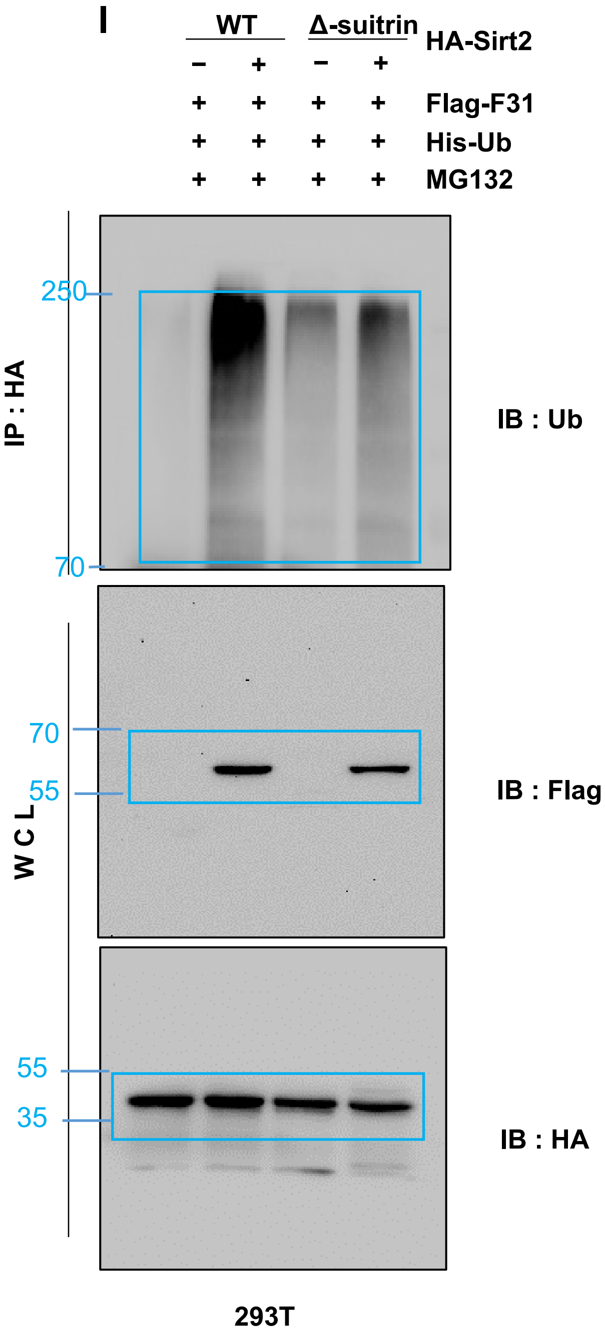

J

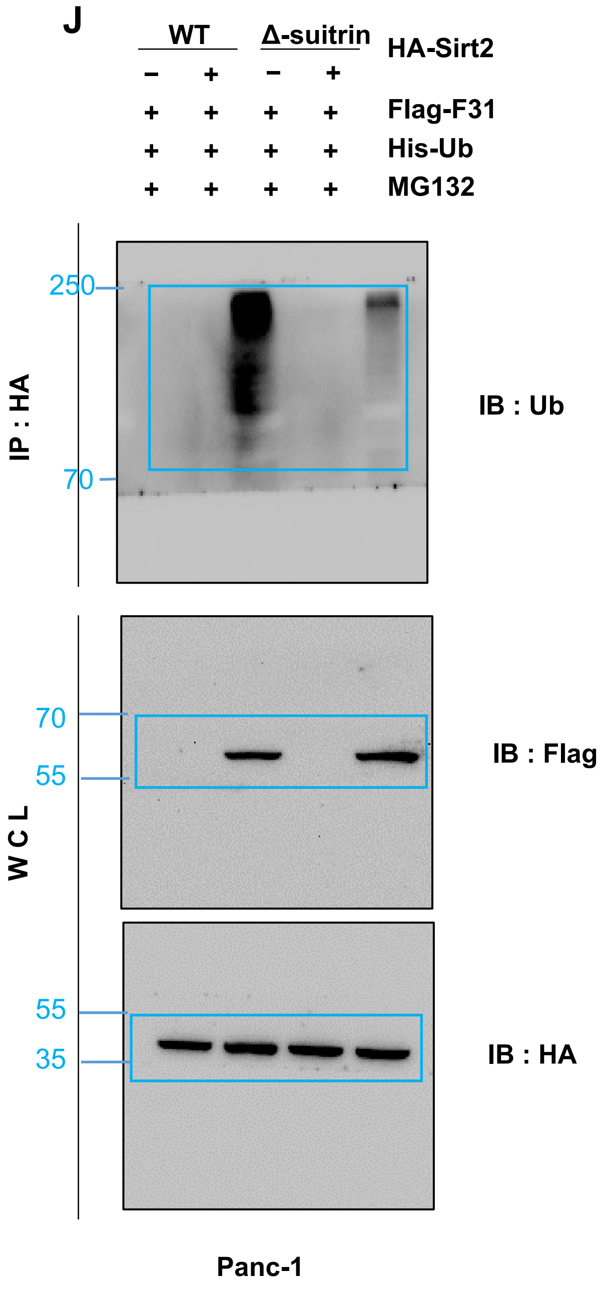

K

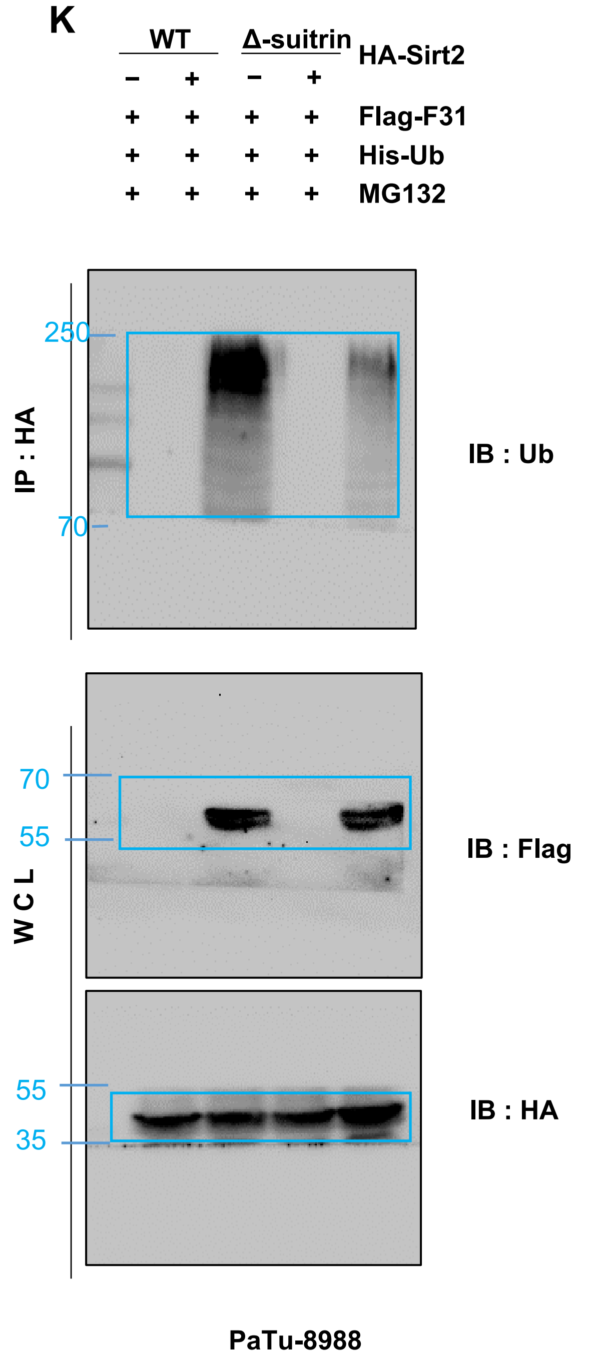

Fig7

A

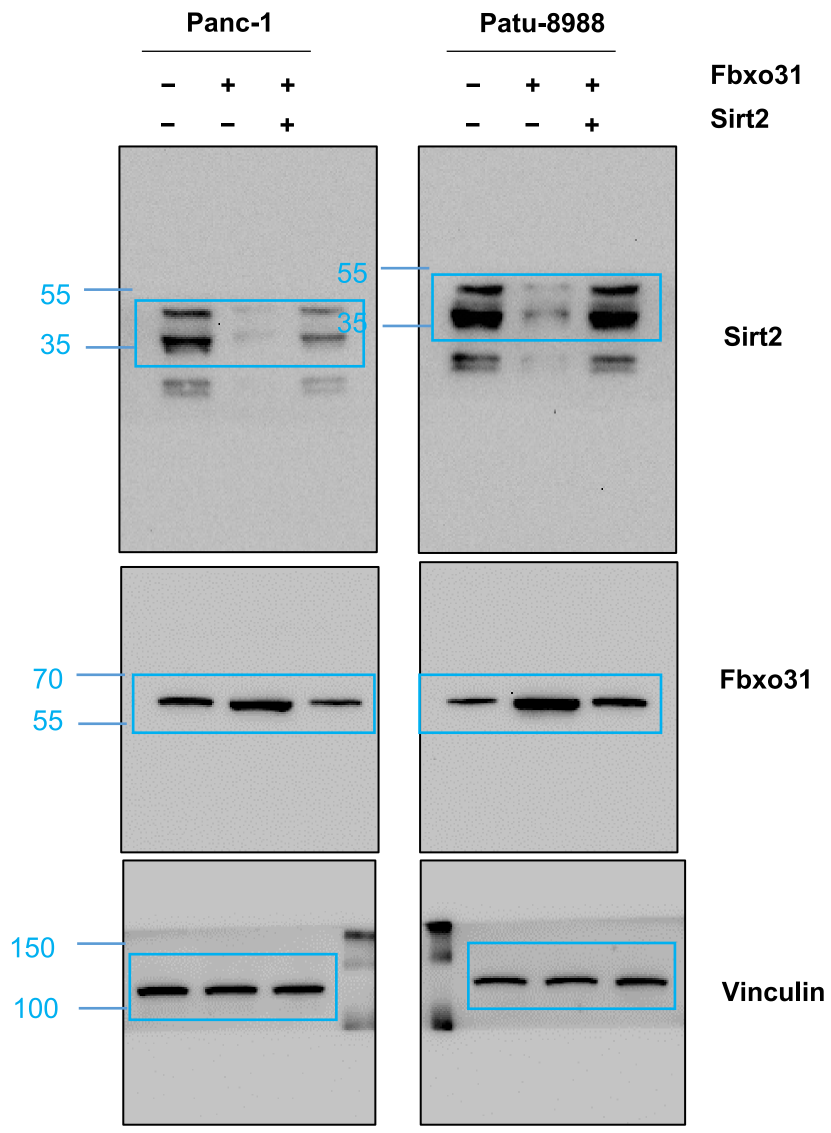

Fig 8

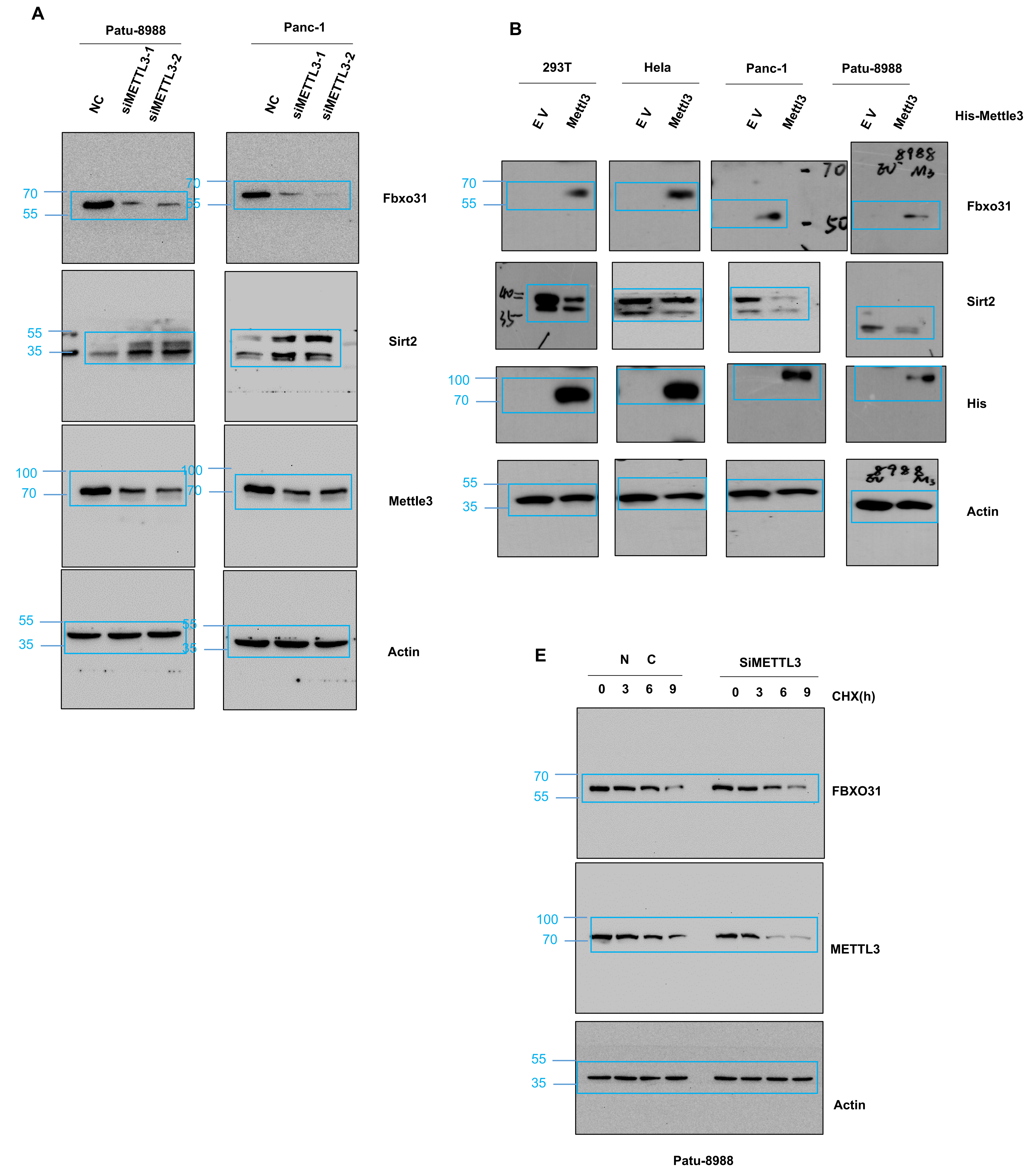
A
Patu-8988
Panc-1
NC
siMETTL3-1
siMETTL3-2
Fbxo31
Sirt2
Mettle3
Actin
70
55
35
100
B
293T
Hela
Panc-1
Patu-8988
E V
Mettl3
His-Mettle3
Fbxo31
Sirt2
His
Actin
E
N C
SiMETTL3
0 3 6 9
CHX(h)
FBXO31
METTL3
Actin
Patu-8988

G

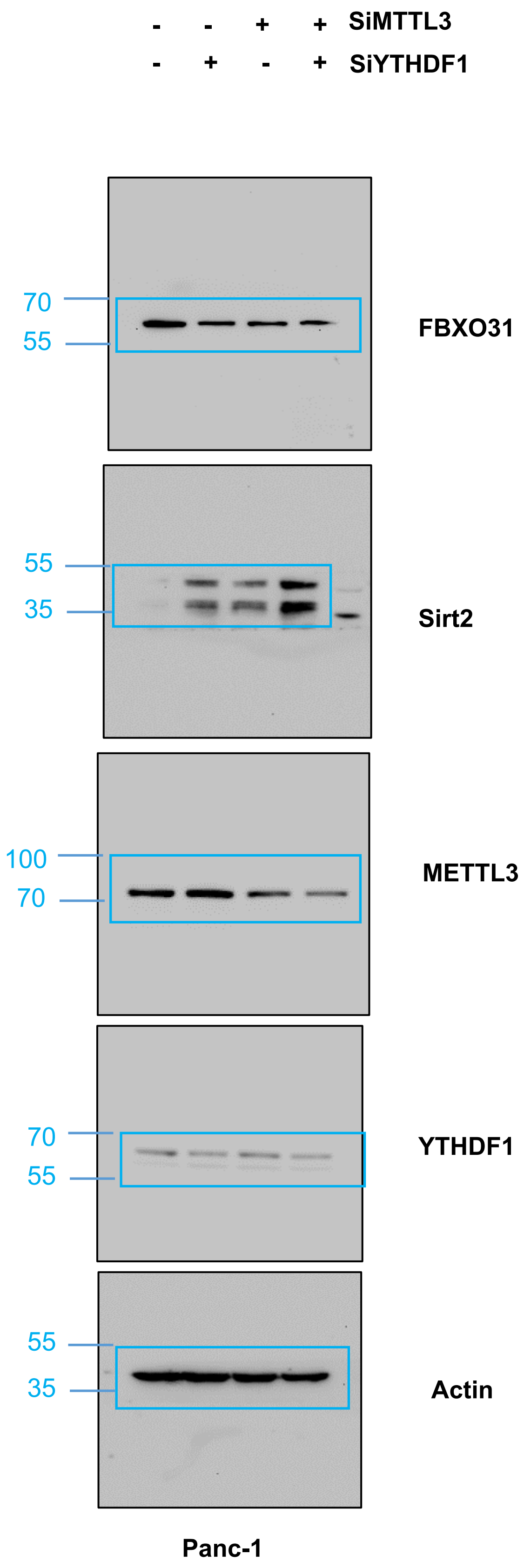

- - + + SiMTTL3
- + - + SiYTHDF1
70
55
FBXO31
55
35
Sirt2
100
70
METTL3
70
55
YTHDF1
55
35
Actin
Panc-1

H

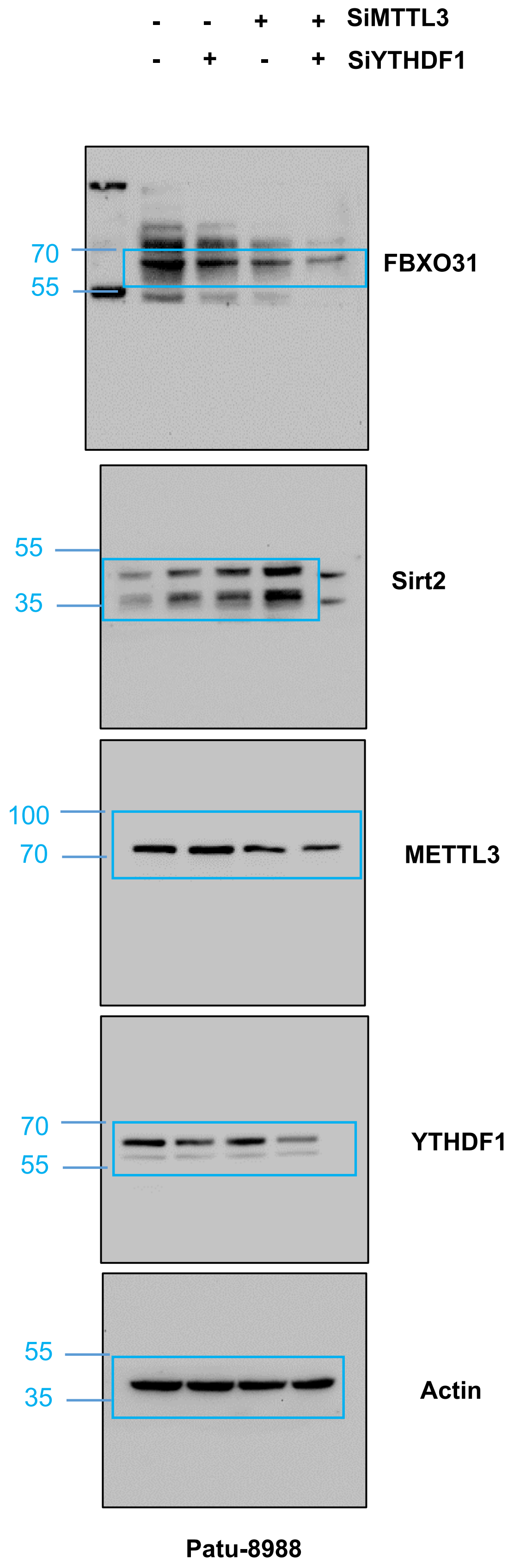

- - + + SiMTTL3
- + - + SiYTHDF1
70
55
FBXO31
55
35
Sirt2
100
70
METTL3
70
55
YTHDF1
55
35
Actin
Patu-8988

Fig S2

D

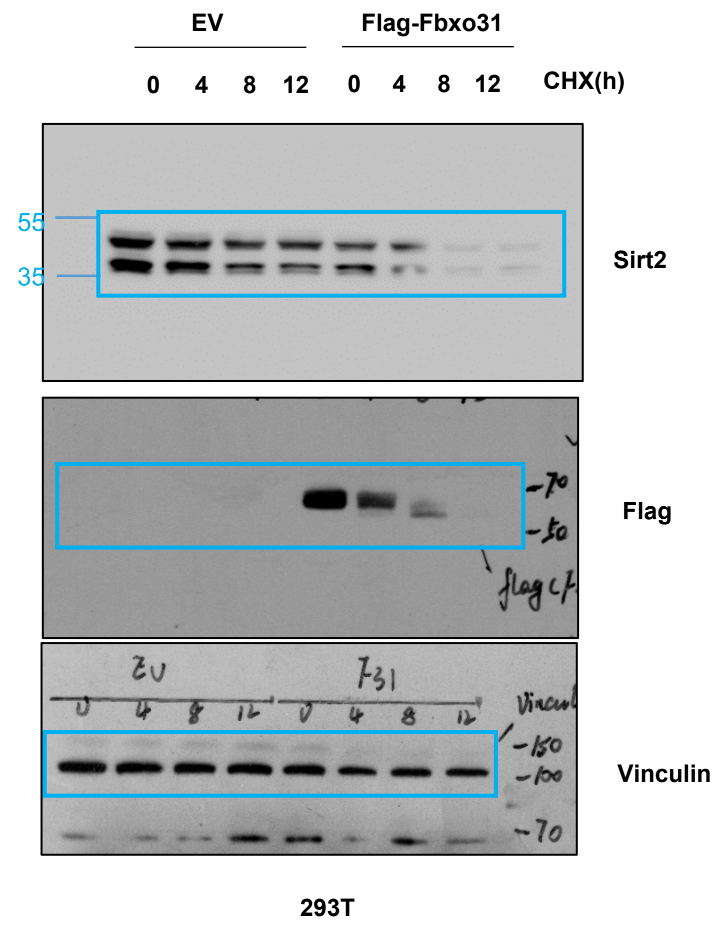
EV
Flag-Fbxo31
0 4 8 12 0 4 8 12 CHX(h)
55
35
Sirt2
Flag
Vinculin
293T

F

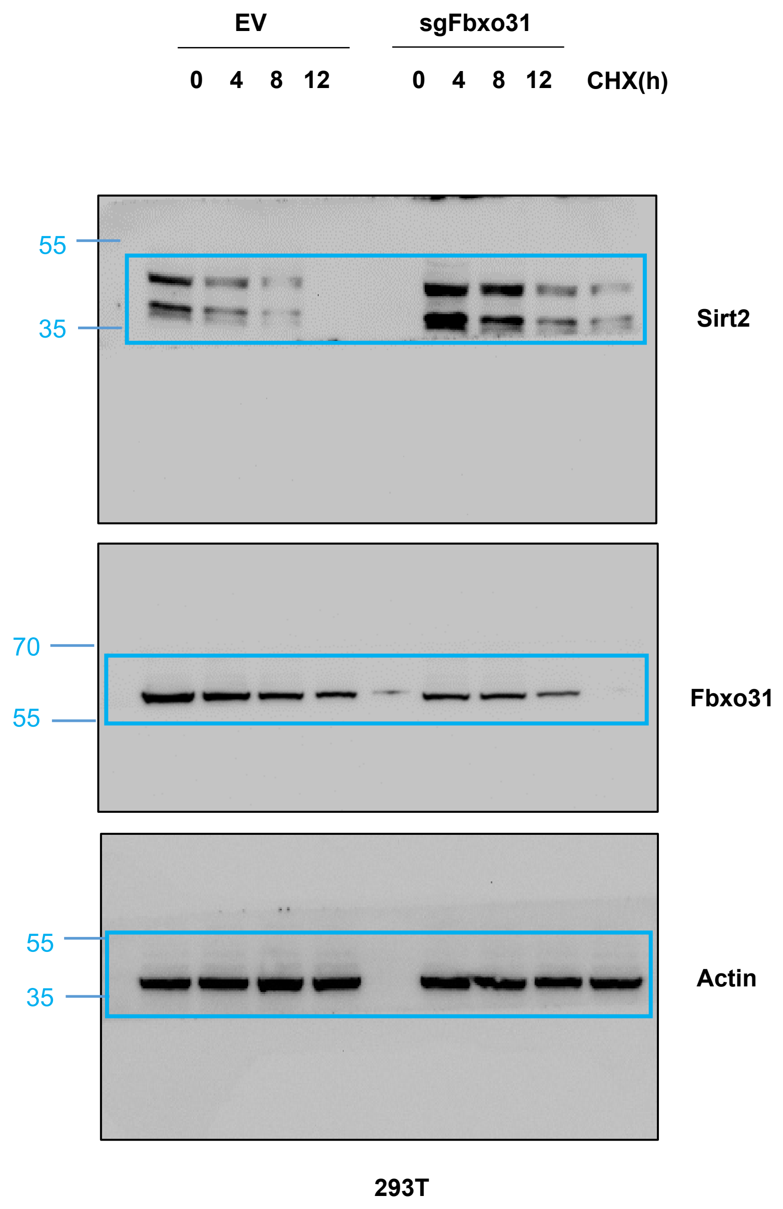
EV
sgFbxo31
0 4 8 12 0 4 8 12 CHX(h)
55
35
Sirt2
70
55
Fbxo31
55
35
Actin
293T

Fig S3

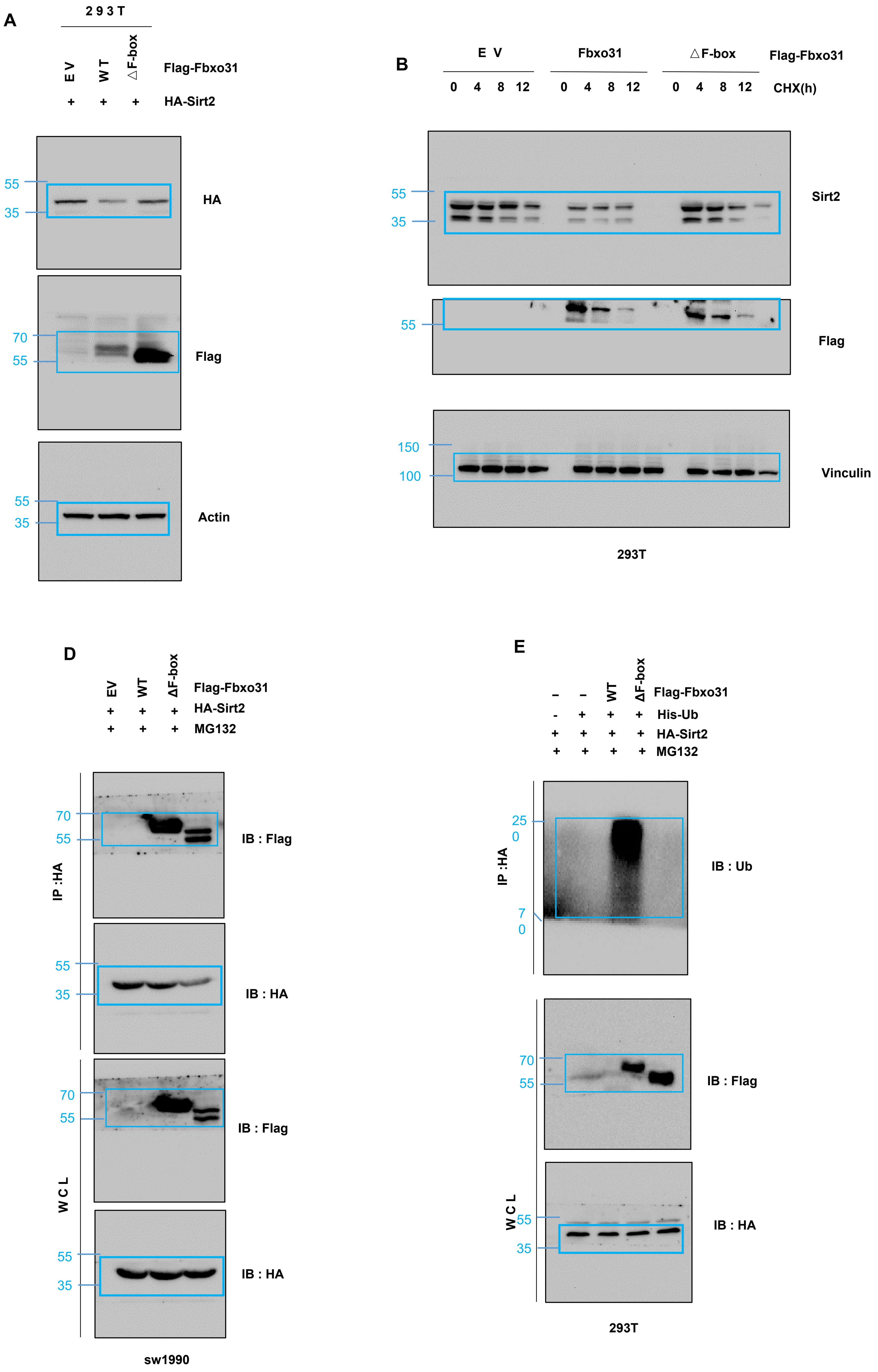

A
293T
EV
WT
△F-box
Flag-Fbxo31
+ + +
HA-Sirt2
55
35
HA
70
55
Flag
55
35
Actin
B
EV
Fbxo31
△F-box
Flag-Fbxo31
0 4 8 12
0 4 8 12
0 4 8 12
CHX(h)
55
35
Sirt2
55
Flag
150
100
Vinculin
293T
D
EV
WT
ΔF-box
Flag-Fbxo31
+ + +
HA-Sirt2
+ + +
MG132
IP :HA
70
55
IB : Flag
55
35
IB : HA
WCL
70
55
IB : Flag
55
35
IB : HA
sw1990
E
– – WT ΔF-box
Flag-Fbxo31
- + + +
His-Ub
+ + + +
HA-Sirt2
+ + + +
MG132
IP :HA
250
70
IB : Ub
70
55
IB : Flag
WCL
55
35
IB : HA
293T

Fig S4

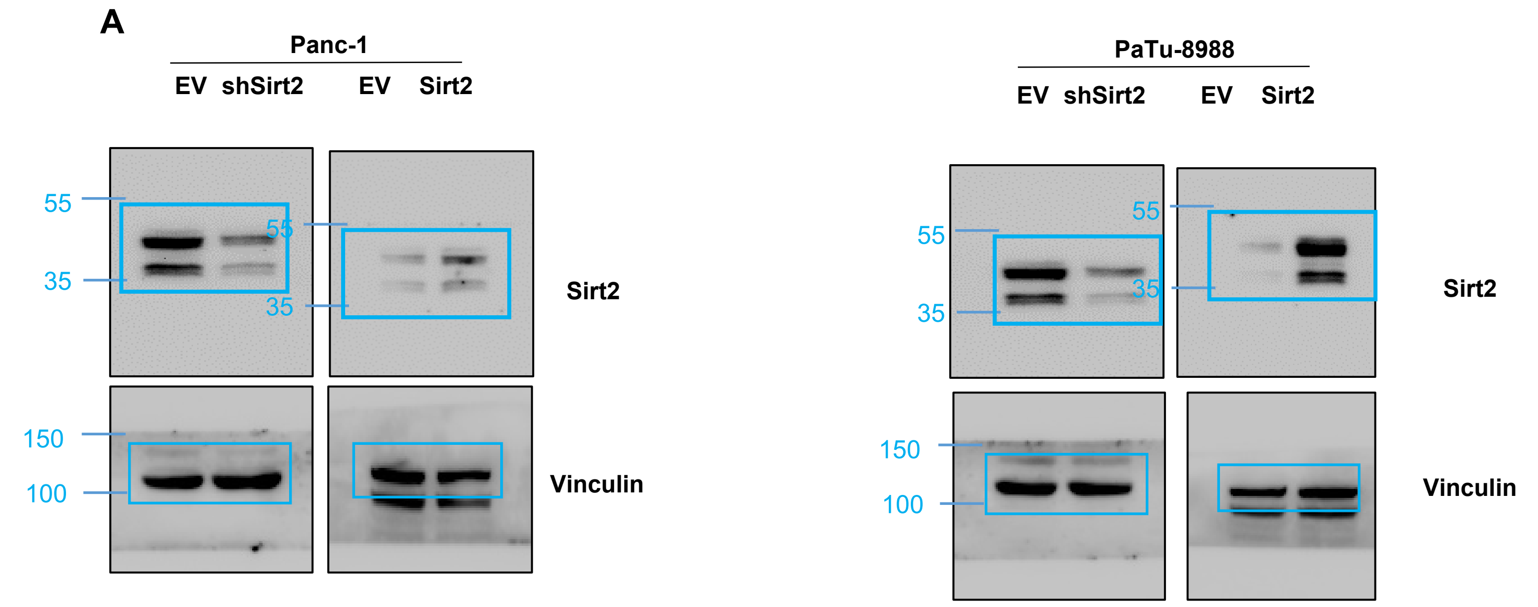

Fig S5 D

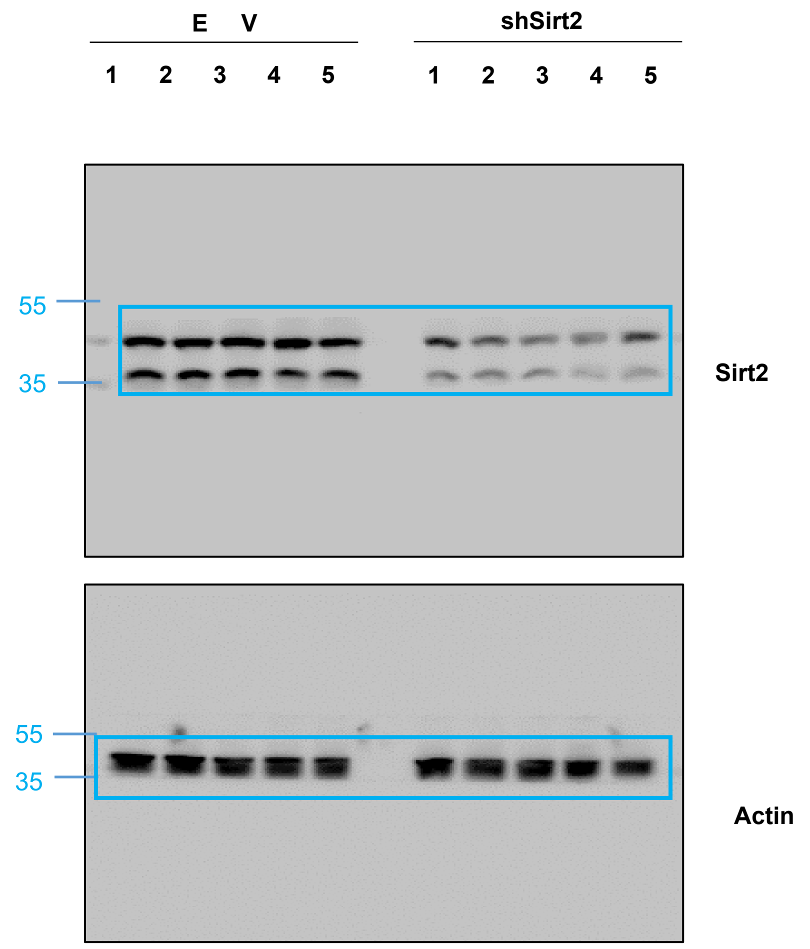

FigS6

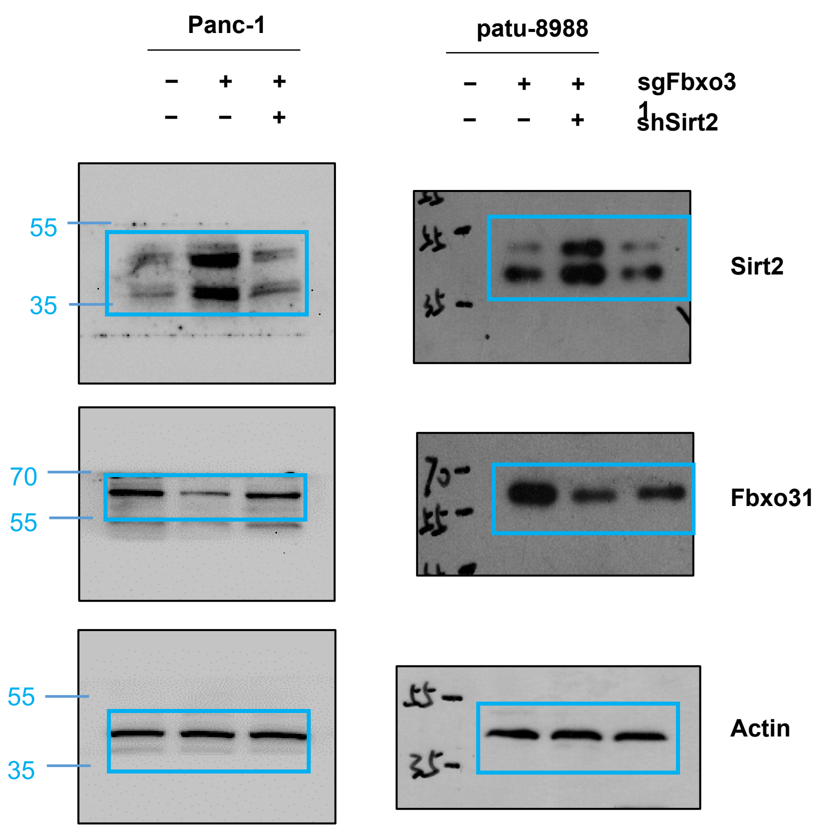

Fig S9

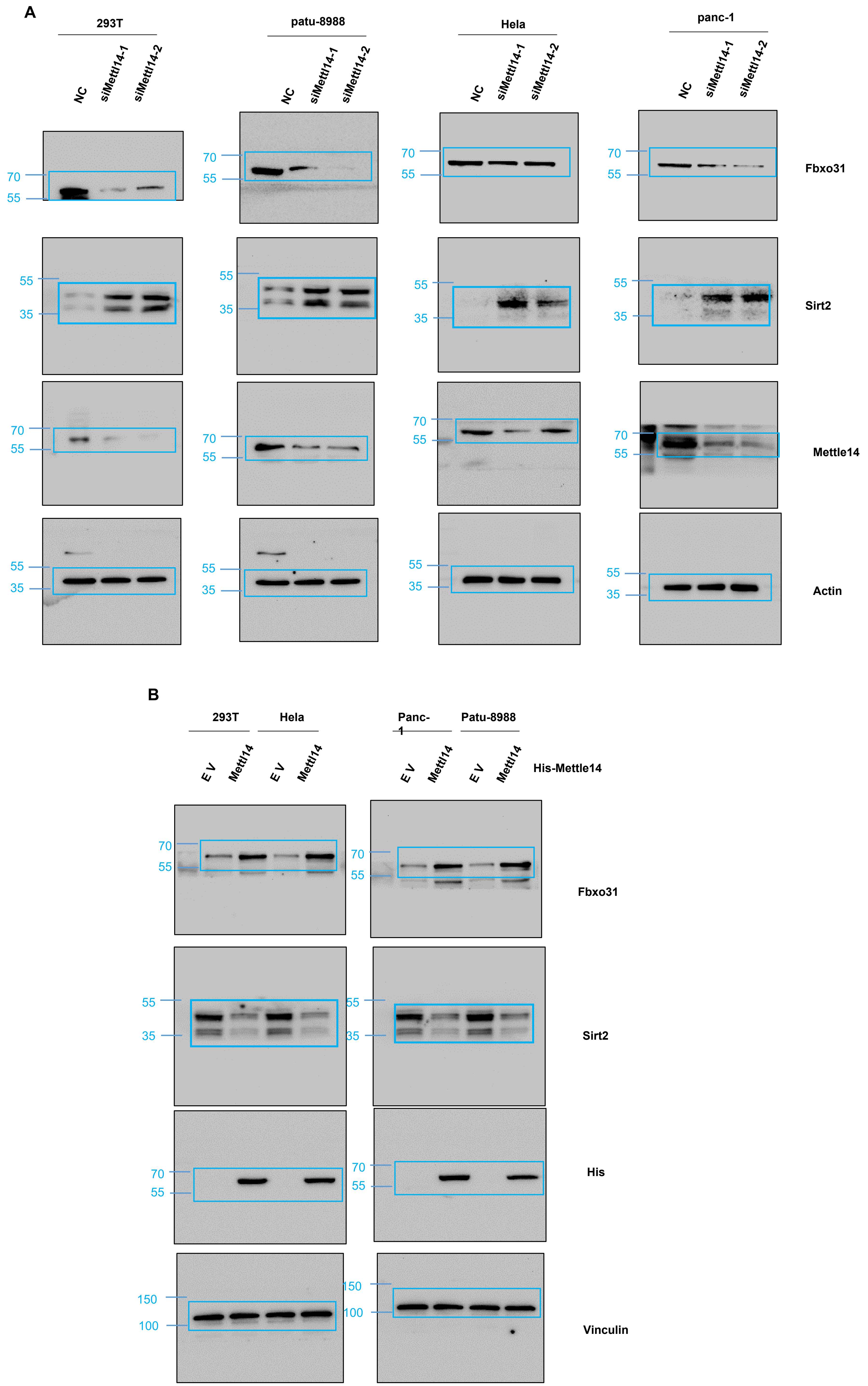
A
293T
patu-8988
Hela
panc-1
NC
siMettl14-1
siMettl14-2
70
55
35
Fbxo31
Sirt2
Mettle14
Actin
B
293T
Hela
Panc-1
Patu-8988
E V
Mettl14
His-Mettle14
150
100
Fbxo31
Sirt2
His
Vinculin
